# Complementary roles of dorsal and ventral hippocampus in the flexible adaptation of goal-directed behavior

**DOI:** 10.1126/sciadv.adx7514

**Published:** 2025-11-28

**Authors:** Maryam Hasantash, Yifei Li, Arturo Torres-Herraez, Christoph Kellendonk, Christoph Anacker

**Affiliations:** ^1^ Department of Psychiatry, Division of Systems Neuroscience, Columbia University, New York, NY, USA.; ^2^ Research Foundation for Mental Hygiene, New York State Psychiatric Institute, New York, NY, USA.; ^3^ Department of Psychiatry, Division of Molecular Therapeutics, Columbia University, New York, NY, USA.; ^4^ Department of Molecular Pharmacology and Therapeutics, Columbia University, New York, NY, USA.; ^5^ Columbia Institute for Developmental Sciences, New York, NY, USA.; ^6^ Columbia University Stem Cell Initiative (CSCI), New York, NY, USA.

## Abstract

The ability to adapt previously learned behaviors is crucial for survival in dynamically changing environments. The hippocampus has been implicated in associative learning, but how hippocampus activity along its septotemporal axis contributes to flexible adaptation is unknown. Using in vivo Ca^2+^ recordings and functional inhibition of dorsal CA1 (dCA1) and ventral CA1 (vCA1) neurons in mice during complementary cognitive flexibility tasks, we find that dCA1 is engaged and functionally required during consolidation of a learned contingency both before and after a rule change, whereas vCA1 is uniquely recruited during, and necessary for, early adaptation to a new contingency. This vCA1-dependent adaptation relies on a perseverative error signal, which is encoded in vCA1 and required for behavior updating. These results highlight a previously unknown division of labor within the hippocampus, in which vCA1 enables flexible adaptation when mismatches in expected and actual outcomes are detected, whereas dCA1 stabilizes newly learned information.

## Teaser

vCA1 drives flexible adaptation to new rules by detecting perseverative error signals, while dCA1 stabilizes learned information.

## INTRODUCTION

“It is not what happens to you, but how you react to it that matters.” These words by the philosopher Epictetus highlight the importance of adapting our behavior in response to changes in our environment. Although this wisdom is almost 2000 years old, we still do not fully understand the neurobiological mechanisms that control how individuals flexibly adjust goal-directed behavior when environmental demands change.

Cognitive flexibility is a fundamental aspect of adaptation and essential for navigating dynamic environments in which food availability and predator threats fluctuate and require animals to adjust foraging behavior, shelter-seeking strategies, and predator avoidance tactics based on changing features in the environment. In humans, cognitive flexibility facilitates goal-directed decision-making and is associated with greater psychological well-being, likely due to a higher propensity for adapting effectively to new situations. Cognitive flexibility deficits, on the other hand, are often associated with greater perseveration, rumination, and habitual behavior, which are common symptoms of mental disorders and dementia, and can exacerbate emotional distress (*1*–*3*).

Flexible behavior adaptation requires multiple aspects of cognition, including learning of an initial action-outcome contingency, detecting a change in the contingency, and learning to consistently respond to the new contingency. Traditionally, prefrontal brain regions have been most strongly implicated as neural substrates for flexible cognition due to their role in action planning, decision-making, and working memory (*4*–*6*). However, considering growing insight into the vast cognitive repertoire required for behavior adaptation, our knowledge of neurobiological mechanisms involved in diverse aspects of cognitive flexibility is rapidly increasing.

The hippocampus is known for its role in associative learning (*7*, *8*), memory-guided behaviors (*9*), and in context-dependent changes in goal-directed behavior (*10*), which are fundamental functions that enable an individual to adjust its behavior based on past experiences. Although human studies have shown that hippocampal gray matter volume correlates with reversal learning performance and that amnesic patients with hippocampal lesions make more perseverative errors in reversal tasks (*11*–*15*), a direct functional role for the hippocampus in cognitive flexibility has never been examined.

The hippocampus is functionally heterogeneous along its septotemporal axis. In rodents, the dorsal pole of the hippocampus has primarily been implicated in spatial navigation and learning (*16*, *17*), whereas the ventral hippocampus is important for goal-directed behaviors (*18*), motivational salience (*19*–*21*), associative learning (*7*, *22*–*25*), and affective states (*26*–*28*). The hippocampus also responds to novelty (*29*), which, in turn, facilitates new learning by modulating neural activity specifically in the ventral hippocampus (*30*). Mechanistic research into the role of the hippocampus in cognitive flexibility has focused on how neuroplasticity, such as adult hippocampal neurogenesis, contributes to flexible learning (*26*, *31*). Although this type of plasticity reflects structural changes that enable long-term adaptation, little is known about how dynamic responses in neural activity along the hippocampus septotemporal axis contribute to different stages of reversal learning. Understanding the functional interactions between rapid adjustments in task-specific dorsal and ventral hippocampus activity and behavior will be crucial to develop a comprehensive understanding of how the hippocampus contributes to behavior adaptation in dynamic environments.

In this study, we used in vivo calcium (Ca^2+^) recordings and chemogenetic inhibition of dorsal CA1 (dCA1) and ventral CA1 (vCA1) in freely moving mice during three cognitive flexibility tasks. This approach allowed us to determine dynamic neural response profiles and functional contributions of these two hippocampal subregions to complementary aspects of cognitive flexibility, including learning of an initial action-outcome contingency, perseverative error detection, behavior updating, and the sustained adaptation of new behavioral response patterns.

## RESULTS

### Complementary dCA1 and vCA1 neural activity dynamics during reversal learning

**Fig. 1.**
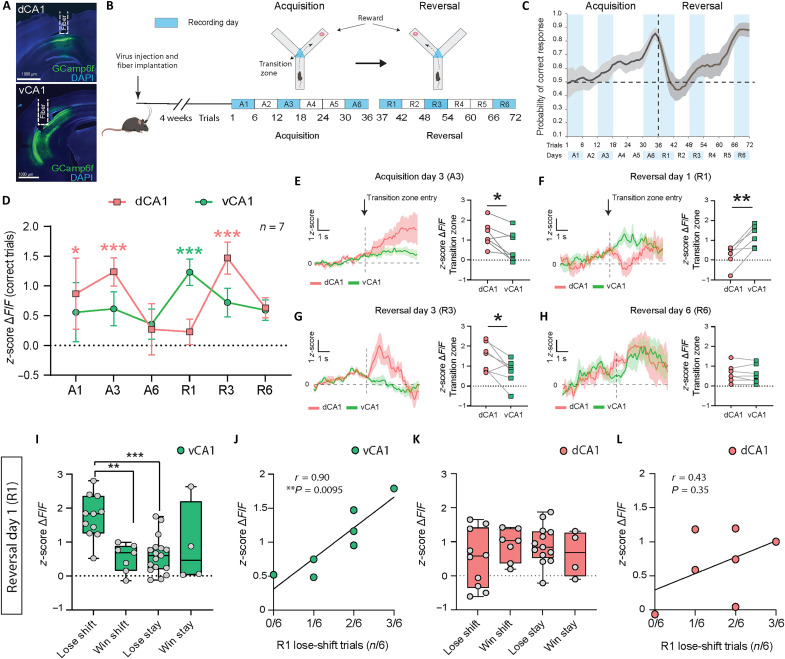
Simultaneous Ca^2+^ recordings show vCA1 recruitment during lose-shift adaptation and dCA1 recruitment during behavior consolidation. (**A**) Fiber placement in dCA1 and vCA1. (**B**) Y maze schematic indicating the transition zone in which Ca^2+^ signals were quantified. Experimental timeline indicates recording days and trials (A1 to A6, acquisition days; R1 to R6, reversal days). (**C**) State-space model learning curve. Recording days are indicated in blue. (**D**) Ca^2+^ activity during the 3-s period after entering the transition zone during correct-choice trials on acquisition and reversal days. Two-way RM ANOVA: Region × Time, *F*(10,58) = 2.5, *P = 0.014; Region, *F*(2,12) = 23.6, ***P < 0.001; Time, *F*(5,30) = 0.79, *P* = 0.57; *n*_dCA1_ = 7 mice, *n*_vCA1_ = 7 mice. Compared to baseline, dCA1 activity (red) increased on A1 (*P = 0.014), A3 (***P = 0.0003), and R3 (***P < 0.0001). vCA1 activity (green) increased on R1 (***P = 0.0007). (**E** to **H**) Ca^2+^ signals aligned to transition zone entry. Left: Average *z*-scored Ca^2+^ traces. Right: Average Ca^2+^ signal per mouse (*z*-scored Δ*F*/*F* over a 3-s period upon entering the transition zone). Paired *t* test: (E) A3 (*P = 0.03), *n* = 7. (F) R1 (**P = 0.004), *n* = 6 (one mouse did not make any correct choice on R1). (G) R3 (*P = 0.03), *n* = 7. (H) R6 (*P* = 0.198), *n* = 7. (**I**) Average vCA1 Ca^2+^ activity for strategy use on R1 [one-way ANOVA, *F*(3,35) = 8.085, ***P = 0.0003, Tukey post hoc test: Lose shift versus Win shift, **P = 0.002; Lose shift versus Lose stay, ***P = 0.0004; linear mixed-effects model with “mouse” as a random effect: main effect of strategy, **P = 0.002; Lose shift versus Win shift, β = −1.07, **P = 0.002; Lose shift versus Lose stay, β = −1.19, ***P < 0.0001; leave-one-out analysis confirmed that these effects were not driven by any single mouse, **P < 0.01 for all comparisons]. (**J**) vCA1 activity correlates with lose-shift trials on R1 (*r* = 0.9, **P = 0.0095). (**K**) Average dCA1 Ca^2+^ activity on R1 showed no significant differences across strategies [*F*(3,30) = 1.43, *P* = 0.25]. (**L**) dCA1 activity did not correlate with lose-shift trials on R1 (*r* = 0.43, *P* = 0.35). *N* = 7 mice. Means ± SEM.

Reversal learning—the capacity to adapt a response from a previously relevant contingency to a new contingency—is a key component of cognitive flexibility. To determine how the hippocampus contributes to reversal learning, we injected the neural activity-dependent Ca^2+^ sensor, GCamp6f, into dCA1 and vCA1 and used in vivo fiber photometry to simultaneously record Ca^2+^ activity from each region (Fig. 1A and fig. S1). We first recorded mice in a deterministic Y maze task in which one arm always contained a reward (sweet cereal powder) whereas the other arm did not contain a reward. After learning the initial arm-reward contingency over 36 trials for 6 days (6 trials per day), the reward was switched to the previously unrewarded arm and mice needed to learn the change in the arm-reward contingency during a reversal learning period for an additional 36 trials over 6 days (Fig. 1B). We used a state-space model to capture learning dynamics by estimating trial-by-trial changes in the probability of a correct response based on mice’ choice behavior (*32*). This model showed that mice learned the initial arm-reward contingency with ~90% accuracy by the end of the acquisition period (trial 36) and were able to learn the reversed contingency to the same level of accuracy during the subsequent reversal phase (until trial 72; Fig. 1C).

We recorded dCA1 and vCA1 Ca^2+^ activity on 6 days spanning the initial learning and reversal phases of the task: acquisition days A1 (early learning, trials 1 to 6), A3 (immediate learning, trials 13 to 18), and A6 (late learning, trials 30 to 36) and reversal days R1 (early reversal learning, trials 37 to 42), R3 (immediate reversal learning, trials 49 to 54), and R6 (late reversal learning, trials 67 to 72). We then analyzed Ca^2+^ responses during a 3-s period when mice transitioned into the correct arm on each recording day because we reasoned that the period during which mice transition into the correct arm would reflect the animal’s choice.

vCA1 was largely inactive during correct arm transitions on acquisition days A1 to A6 but vCA1 activity increased following the contingency reversal on the first reversal day (R1) and remained slightly elevated on the later reversal days (Fig. 1, D to H, green line). In contrast, dCA1 responded during correct arm transitions when sustained learning of the initial rule was required on days A1 and A3 and similarly when sustained learning of the reversed reward location was required on reversal day R3 (Fig. 1, D to H, red line). Whereas vCA1 responses were specific to transitions into the correct arm after reversal, dCA1 activity increased during transitions into either the correct or the incorrect arm during acquisition and reversal learning (fig. S2). We therefore tested whether the specific recruitment of vCA1 during correct choices following the rule reversal would be related to the use of adaptive strategies that lead to correct choice behavior. Following the rule reversal on R1, vCA1 activity in the transition zone specifically increased during lose-shift behaviors (Fig. 1I), and activity across all trials on R1 correlated with the number of times each mouse used a lose-shift strategy (Fig. 1J). This association between neural activity and lose-shift strategy use was not seen in dCA1 on the first reversal day (R1; Fig. 1, K and L) but emerged during sustained learning of the new contingency on the third reversal day (R3; fig. S3D).

Because increased vCA1 activity during lose-shift suggests that previous incorrect outcomes influence neural responses during subsequent correct choices, we next tested whether decision-related activity is associated with outcome-related activity from the prior trial. To this end, we quantified vCA1 activity during a 3-s period immediately following reward consumption on correct trials and when mice reach the end of the empty arm without finding the reward on incorrect trials. On reversal day R1, vCA1 activity was higher following lack of reward attainment than following reward consumption and correlated with vCA1 activity during subsequent correct choices (Ca^2+^ activity in the transition zone), indicating an association between error-related and subsequent choice-related signals in vCA1 (fig. S4, A to C). We did not find this association in dCA1 (fig. S4D).

Together, these data indicate that vCA1 is recruited specifically during adaptation to a contingency change (i.e., which arm contains reward), whereas dCA1 responds during consolidation of new information both before and after a contingency change.

### dCA1 and vCA1 exert distinct but complementary control over reversal learning

**Fig. 2.**
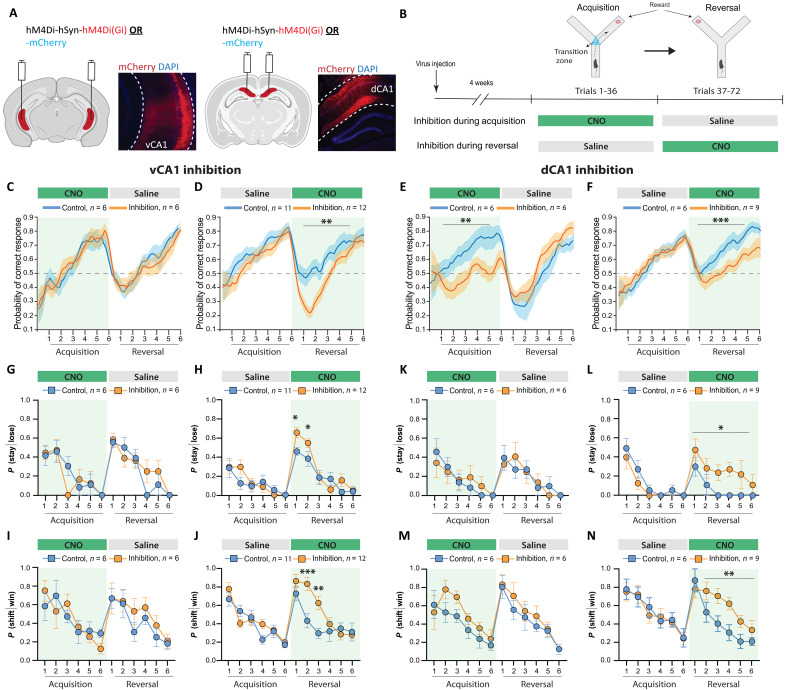
Region-specific inhibition reveals complementary roles of vCA1 and dCA1 for flexible behavioral adaptation and consolidation. (**A**) Virus injections in vCA1 and dCA1. (**B**) Experimental design for vCA1 or dCA1 inhibition during acquisition or reversal in the Y maze. (**C** to **F**) State-space model learning curves. (C) vCA1 inhibition during acquisition did not affect learning or reversal learning performance (*n*_control_ = 6, *n*_hM4Di_ = 6). (D) vCA1 inhibition after reversal impaired reversal learning [Inhibition × Time, *F*(35,735) = 2.176, ***P = 0.0001; Inhibition, *F*(1,21) = 19.48, **P = 0.002; Time, *F*(35,735) = 23.93, ***P < 0.0001; *n*_control_ = 11, *n*_hM4Di_ = 12]. (E) dCA1 inhibition during acquisition impaired initial learning [Inhibition × Time, *F*(35,350) = 1.038, *P* = 0.414; Inhibition, *F*(1,10) = 19.75, **P = 0.0012; Time, *F*(35,350) = 2.819, ***P < 0.0001, *n*_control_ = 6, *n*_hM4Di_ = 6]. (F) dCA1 inhibition after reversal impaired reversal learning [Inhibition × Time, *F*(35,455) = 1.17, *P* = 0.23; Inhibition, *F*(1,13) = 35.24, ***P < 0.0001; Time, *F*(35,455) = 6.49, ***P < 0.0001; *n*_control_ = 6, *n*_hM4Di_ = 9]. (**G**) vCA1 inhibition during acquisition did not affect *P*(stay|lose). *n*_control_ = 6, *n*_hM4Di_ = 6. (**H**) vCA1 inhibition during reversal increased *P*(stay|lose) immediately after reversal [Inhibition × Time, *F*(5,123) = 2.29, *P < 0.05; Inhibition, *F*(1,123) = 3.89, *P* = 0.05; Time, *F*(5,123) = 29.66, ***P < 0.001; Tukey post hoc test, R1: *P = 0.01, R2: *P = 0.04; *n*_control_ = 11, *n*_hM4Di_ = 12]. (**I**) vCA1 inhibition during acquisition did not affect *P*(shift|win). *n*_control_ = 6, *n*_hM4Di_ = 6. (**J**) vCA1 inhibition during reversal increased *P*(shift|win) [Inhibition × Time, *F*(5,125) = 3.31, **P = 0.008; Inhibition, *F*(1,125) = 11.11, **P = 0.001; Time, *F*(5,125) = 14.43, ***P < 0.0001; Tukey’s post hoc test: R2, ***P = 0.0002; R3, **P = 0.002; *n*_control_ = 11, *n*_hM4Di_ = 12]. (**K**) dCA1 inhibition during acquisition did not affect *P*(stay|lose). *n*_control_ = 6, *n*_hM4Di_ = 6. (**L**) dCA1 inhibition during reversal increased *P*(stay|lose) during the late phase of reversal learning [Inhibition × Time, *F*(5,77) = 0.16, *P* = 0.98; Inhibition, *F*(1,77) = 11.51, *P = 0.001; Time, *F*(5,77) = 2.75, *P = 0.025; *n*_control_ = 6, *n*_hM4Di_ = 9]. (**M**) dCA1 inhibition during acquisition did not affect *P*(shift|win). *n*_control_ = 6, *n*_hM4Di_ = 6). (**N**) dCA1 inhibition during reversal increased *P*(shift|win) during the late phase of reversal [Inhibition × Time, *F*(5,65) = 1.35, *P* = 0.26; Inhibition, *F*(1,13) = 14.38, **P = 0.002; Time, *F*(5,65) = 9.97, ***P < 0.0001; *n*_control_ = 6, *n*_hM4Di_ = 9]. Means ± SEM.

To determine the functional roles of dCA1 and vCA1 for reversal learning, we injected mice bilaterally with the inhibitory DREADD receptor, hM_4_D_i_, and inhibited each region either during initial rule acquisition or during subsequent reversal learning in the Y maze by injecting the hM_4_D_i_ agonist, clozapine *N*-oxide (CNO; 5 mg/kg) 30 min before the first trial on each day (Fig. 2, A and B, and fig. S5). Consistent with our finding that vCA1 activity increased predominantly following the rule reversal (as shown in Fig. 1, D and F), vCA1 inhibition did not affect initial rule acquisition (Fig. 2C) but impaired reversal learning when inhibition occurred after the contingency change (Fig. 2D). Because our recordings had shown that vCA1 is predominantly recruited during early adaptation to the new rule, whereas dCA1 is engaged during later stages of reversal, we assessed the functional effects of inhibiting each region on early versus late phases of reversal learning. To this end, we fitted a logistic curve to subjects’ choices and compared the slopes of trial-by-trial performance curves between groups as a measure of learning rate. These analyses showed that vCA1 inhibition lowered the probability of making a correct choice on the first reversal day, indicating impaired adaptation to the contingency change, while showing a trend toward a steeper learning curve compared to controls, suggesting faster recovery of learning with continued training (*P* = 0.058; fig. S6D). In contrast, dCA1 inhibition impaired both, initial rule acquisition and reversal learning (Fig. 2, E and F), consistent with its well-established role in learning and memory. dCA1 inhibition impaired continuous learning of both the initial and the new reward location without delaying early adaptation to the new rule immediately after reversal (fig. S6, E and H).

Because we had found that vCA1 activity correlated with lose-shift strategy use after reversal (Fig. 1J), we next tested whether inhibition would affect the use of adaptive behavior strategies. Inhibition of vCA1 during initial acquisition did not alter strategy use (Fig. 2, G and I). However, vCA1 inhibition during reversal increased the probability of repeating a previous incorrect choice and of changing behavior after a previous correct choice during early reversal learning (R1 to R3; Fig. 2, H and J). Inhibition of dCA1 during acquisition did not affect strategy use (Fig. 2, K and M), whereas dCA1 inhibition during reversal increased the probability of repeating a previous incorrect choice and of changing behavior after a previous correct choice during the late phase of reversal learning (R3 to R5; Fig. 2, L and N, and fig. S7).

Collectively, these results indicate distinct but complementary contributions of dCA1 and vCA1 to early versus late phases of learning and adaptation. vCA1 is required to flexibly adjust behavior following a contingency change, which requires the use of adaptive behavior strategies (i.e., lose-shift) to effectively respond to the rule reversal. In contrast, dCA1 is needed to learn and consolidate new information during initial rule acquisition and during learning of a new contingency after an initial, vCA1-dependent adaptation phase.

### vCA1 detects perseverative errors

**Fig. 3.**
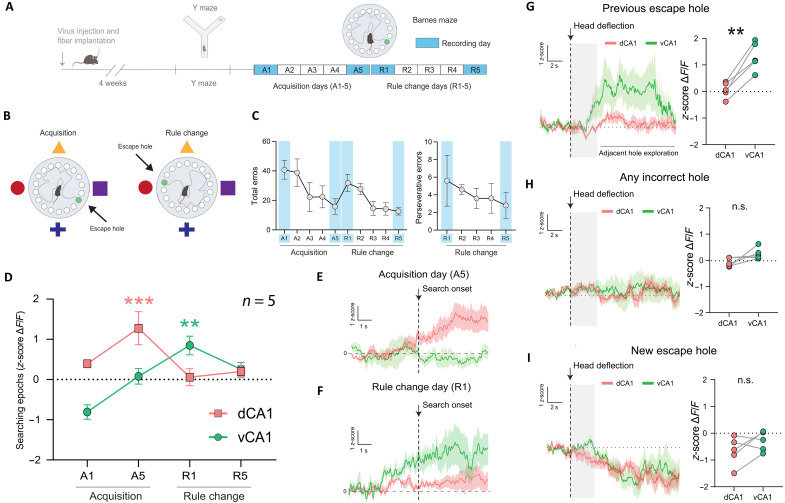
vCA1 is recruited following perseverative errors. (**A**) Experimental timeline for Ca^2+^ recordings in the Barnes maze. Recording days indicated in blue (A1 to A5, acquisition days; R1 to R5, rule change days). (**B**) Barnes maze schematic with spatial room cues and escape hole locations indicated during acquisition (left) and rule change (right). (**C**) Behavioral performance across acquisition and rule change phases. Left: Total errors per day. Right: Perseverative errors per day after rule change. (**D**) Ca^2+^ activity in dCA1 and vCA1 when mice started searching the maze for the escape hole location (*z*-scored Δ*F*/*F* over a 5-s period following search onset). Two-way RM ANOVA: Region × Time, *F*(6,24) = 11.8, ***P < 0.0001; Region, *F*(2,8) = 7.3, *P = 0.016; Time, *F*(3,12) = 5.0, *P = 0.018; *n*_dCA1_ = 5 mice, *n*_vCA1_ = 5 mice. Compared to baseline, dCA1 activity (red line) increased on A5 (Tukey’s post hoc test, ***P < 0.0001) and vCA1 activity (green line) increased on R1 (**P = 0.0023). (**E**) Neural responses aligned to the beginning of search epoch on A5. (**F**) Neural responses aligned to the beginning of search epoch on R1. (**G** to **I**) Neural responses to errors and escape hole detection on R1. Left: Average Ca^2+^ activity traces aligned to head deflections into holes. Right: Pairwise comparison of Ca^2+^ activity between dCA1 and vCA1 for each mouse during the first 15 s of hole investigation. (G) vCA1 activity increased following a perseverative error compared to dCA1 (**P = 0.002). Gray shaded area indicates average head deflection time for all mice. (H) No changes in dCA1 or vCA1 activity during investigation of any other incorrect hole. (**I**) No changes during investigation of the new escape hole. *N* = 5 mice. Means ± SEM. n.s., not significant.

Reversal learning deficits in a binary choice task, such as the Y maze, could result from impairments in learning the new reward location or from perseverative responding to the previous reward location. Because we had seen that vCA1 was necessary for lose-shift behaviors following a contingency reversal in the Y maze (as shown in Fig. 1, I and J), we reasoned that vCA1 would be recruited when mice make a perseverative error (i.e., “lose”) that indicates the need to adjust behavior (i.e., “shift”). To distinguish perseverative from new learning errors, we recorded a subset of the same mice that were recorded in the Y maze in a Barnes maze task in which an escape box was hidden underneath 1 of 20 possible holes lining the circumference of a brightly lit, elevated tabletop. Mice needed to learn the location of the escape hole over a 5-day period after which the box was switched to a new hole located 135° away from the previous escape hole location (Fig. 3, A and B). This task allowed us to assess perseveration versus new learning because, in addition to the previously correct hole, there were 19 possible options for the new target location after the rule change. In contrast to the egocentric nature of the Y maze, the Barnes maze also allowed us to assess neural activity during a spatially demanding allocentric task that requires mice to use spatial cues around the wall of the testing suite to determine the escape hole location. Whereas in the Y maze, the choice arm was blocked off after mice transitioned into the arm, mice were allowed to freely explore the Barnes maze until they found the escape hole location regardless of how many errors were made before reaching the correct hole.

To capture the task-specific need for spatial orientation and based on the assumption that mice decide which hole to target while orientating themselves on the platform, we first quantified overall neural activity when mice started searching the maze for the escape hole location. These “searching” epochs were quantified over a 5-s period when mice initiated searching behavior, either at the beginning of a trial or when traveling back into the center of the platform after exploring holes around the platform perimeter. dCA1 activity increased at the initiation of searching epochs during the acquisition phase, whereas vCA1 activity increased at the initiation of searching epochs on the first reversal day (R1; Fig. 3, D to F). We then investigated neural responses to different types of errors in the Barnes maze on the first reversal day (R1). We found that vCA1, but not dCA1, specifically responded to perseverative errors, defined as head dips into the previous escape hole, and neural activity remained elevated following the perseverative error when mice investigated the holes adjacent to the previous escape hole (Fig. 3G). We did not detect activity changes in either vCA1 or dCA1 following new learning errors when mice investigated any of the other incorrect holes that did not contain the escape box (Fig. 3H). Both dCA1 and vCA1 showed a trend toward decreased activity when mice entered the new escape hole, potentially indicating a reduction in “anxiety-like” behavior or a lower demand on spatial navigation, which would require less vCA1 and dCA1 activity, respectively (Fig. 3I).

### vCA1 inhibition causes perseveration

**Fig. 4.**
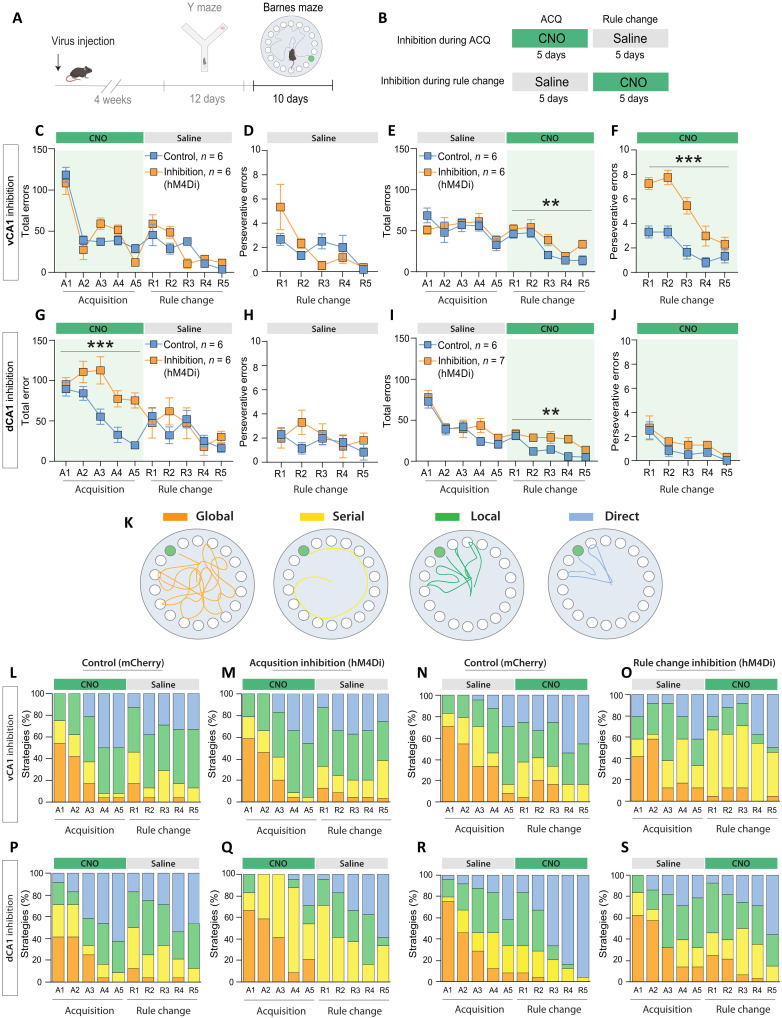
vCA1 inhibition increases perseveration and serial search strategy use. (**A**) Experimental timeline. (**B**) Experimental design for vCA1 or dCA1 inhibition during acquisition or after the rule change in the Barnes maze. (**C**) vCA1 inhibition during acquisition did not affect total errors or (**D**) perseverative errors (*n*_cont_ = 6, *n*_hM4Di_ = 6). (**E**) vCA1 inhibition during the rule change increased total errors [Inhibition × Time, *F*(4,40) = 0.73, *P* = 0.57; Inhibition, *F*(1,10) = 16.40, **P = 0.002; Time, *F*(4,40) = 13.08, ***P < 0.001, *n*_cont_ = 6, *n*_hM4Di_ = 6] and (**F**) perseverative errors [Inhibition × Time, *F*(4,40) = 4.1, **P = 0.008; Inhibition, *F*(1,10) = 40.61, ***P < 0.0001; Time, *F*(4,40) = 24.28, ***P < 0.0001, *n*_cont_ = 6, *n*_hM4Di_ = 6]. (**G**) dCA1 inhibition during acquisition increased total errors [Inhibition × Time, *F*(4,40) = 2.15, *P* = 0.09; Inhibition, *F*(1,10) = 40.33, ***P < 0.0001; Time, *F*(4,40) = 9.33, ***P < 0.001] but (**H**) did not affect perseverative errors during the rule change (*n*_cont_ = 6, *n*_hM4Di_ = 6). (**I**) dCA1 inhibition after the rule change increased total errors [Inhibition × Time, *F*(4,44) = 1.54, *P* = 0.2; Inhibition, *F*(1,11) = 14.21, **P = 0.003; Time, *F*(4,44) = 7.94, ***P < 0.001] but (**J**) did not affect perseverative errors (*n*_cont_ = 6, *n*_hM4Di_ = 7). (**K**) Schematic of behavioral strategies. (**L** to **S**) Proportional use of behavioral strategies during acquisition and reversal phases under vCA1 or dCA1 inhibition. [(L) and (M)] vCA1 inhibition during acquisition did not affect strategy use. [(N) and (O)] vCA1 inhibition during reversal increased serial search strategy use [Inhibition × Time, *F*(4,40) = 0.197, *P* = 0.93; Inhibition, *F*(1,10) = 18.59, **P = 0.0015; Time, *F*(4,40) = 0.78, *P* = 0.54] and decreased local search strategy use [Inhibition × Time, *F*(4,40) = 1.03, *P = 0.4; Inhibition, (*F*(1,10) = 8.32, *P = 0.016; Time, *F*(4,40) = 0.62, *P* = 0.64]. (P and Q) dCA1 inhibition during acquisition increased serial search strategy use [Inhibition × Time, *F*(40,40) = 7.89, ***P < 0.0001; Inhibition, *F*(1,10) = 41.13, ***P < 0.0001; Time, *F*(4,40) = 3.35, *P < 0.0001], decreased direct searching [Inhibition × Time, *F*(4,40) = 2.32, *P* = 0.07; Inhibition *F*(1,10) = 17.41, **P = 0.002; Time, *F*(4,40) = 10.92, ***P < 0.0001], and decreased local searching [Inhibition × Time, *F*(4,40) = 0.83, *P* = 0.51; Inhibition, *F*(1,10) = 10, *P = 0.01; Time, *F*(4,40) = 1.66, *P* = 0.17]. (R and S) dCA1 inhibition during reversal learning reduced direct search strategy use [Inhibition × Time, *F*(4,44) = 3.61, *P = 0.012; Inhibition, *F*(1,11) = 17.66, **P = 0.002; Time, *F*(4,44) = 23.73, ***P < 0.0001]. Means ± SEM.

To determine the functional roles of vCA1 and dCA1 for perseveration and new learning, respectively, we inhibited each region separately during either initial acquisition or during learning after the rule change in the Barnes maze (Fig. 4, A and B). vCA1 inhibition during learning of the initial escape box location did not affect the number of total errors or the number of perseverative errors after the rule change (Fig. 4, C and D), similar to the lack of an effect of vCA1 inhibition on learning the initial reward location in the Y maze. Inhibition during the rule change phase caused a slight but significant increase in the number of total errors (Fig. 4E) and a pronounced increase in the number of perseverative errors (Fig. 4F). In contrast, dCA1 inhibition increased the number of total errors both when inhibition occurred during initial learning or during learning after the rule change, without affecting the number of perseverative errors (Fig. 4, G to J).

Early stages of spatial learning are associated with exploratory search strategies, which become more targeted as learning advances (Fig. 4K). In the Barnes maze, we find that mice used mostly exploratory search strategies (global and serial searches) during initial learning on days A1 and A2 and progressively used more targeted spatial strategies (local and direct searches) toward the end of the learning phase on days A4 and A5 (Fig. 4, L to S).

Inhibiting vCA1 during acquisition did not affect strategy use (Fig. 4, L and M). However, when we inhibited vCA1 after the rule change, mice increased their use of exploratory, serial search strategies at the expense of targeted, local searches (Fig. 4, N and O; yellow bars: serial searches; green bars: local searches), indicating that, in line with increased errors following the rule change, inhibition impaired goal-directed learning strategies when mice needed to adapt their behavior following the detection of a perseverative error. Accordingly, when we measured the time and distance traveled to reach the target hole location, we found that vCA1 inhibition after the rule change increased both the latency and the path length to the new escape hole, likely reflecting the increased use of exploratory as compared to targeted search strategies (fig. S8, B and F).

Mice with dCA1 inhibition during acquisition continued using predominantly exploratory search strategies during the acquisition phase, especially serial searching, possibly due to an impaired ability to learn the spatial configuration of the task when dCA1 was taken offline (Fig. 4, P and Q; yellow bars). Accordingly, dCA1 inhibition also increased the latency and the distance traveled to reach the escape hole location during initial acquisition (fig. S8, C and G). When we inhibited dCA1 following the change in the escape hole location, mice showed a reduction in their ability to use direct spatial search strategies at the expense of serial searching and local searches on the later days of reversal learning (R3 to R5; Fig. 4, R and S, and fig. S8, D and H).

Together, these findings indicate that vCA1 detects perseverative errors and that this detection of a mismatch between expected and actual outcomes in vCA1 is functionally important to overcome perseveration and to facilitate goal-directed search strategies during adaptation to a contingency change. In contrast, dCA1 is important for learning a contingency based on spatial cues and for using goal-directed spatial search strategies both before and after a rule change.

### vCA1 perseverative error signals predict choice behavior during nonspatial reversals and extradimensional set shifts

**Fig. 5.**
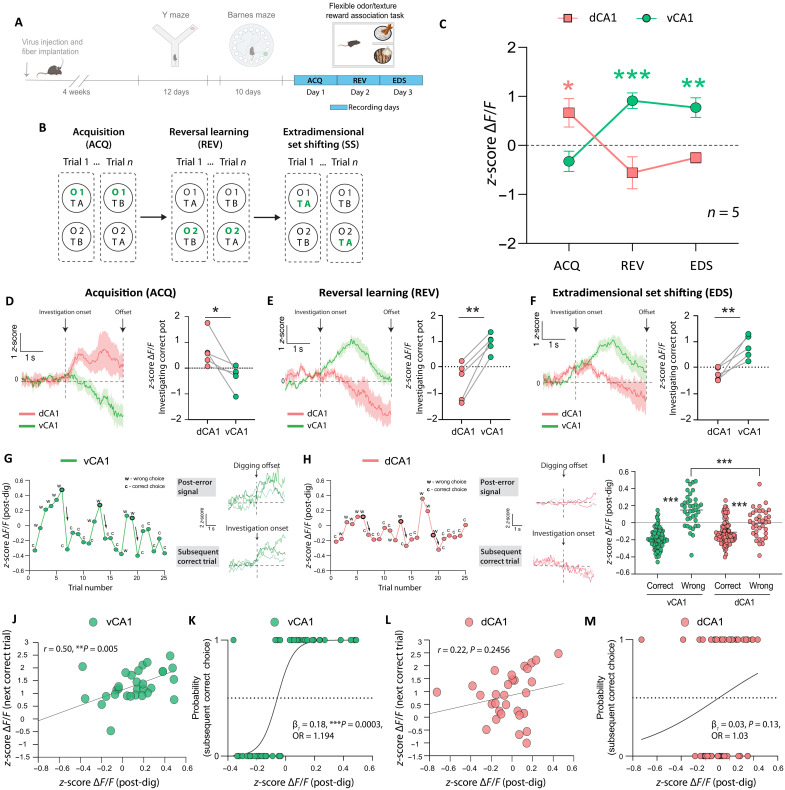
Perseverative error signals in vCA1 predict subsequent correct choices. (**A**) Time. (**B**) Schematic. (**C**) Average Ca^2+^ activity on correct trials on acquisition (ACQ), reversal (REV), and extradimensional set shift (EDS) days. Two-way RM ANOVA: Region × Time, *F*(2,8) = 19.74, ***P = 0.0008; Region, *F*(1,5) = 15.65; *P = 0.017; Time, *F*(2,8) = 0.09, *P* = 0.91; *n*_dCA1_ = 5 mice, *n*_vCA1_ = 5 mice. dCA1 activity (red) increased on the ACQ day (*P = 0.035). vCA1 activity (green) increased on REV (***P = 0.0005) and EDS days (**P = 0.0016). (**D** to **F**) Left: Average Ca^2+^ activity traces aligned to correct pot investigation. Right: Pairwise comparison of dCA1 and vCA1 activity. (D) dCA1 activity was higher than vCA1 on the ACQ day (*P = 0.043). (E) vCA1 activity was higher than dCA1 on REV (**P = 0.004) and (F) EDS days (**P = 0.009). (**G**) Left: Example of vCA1 activity during post-digging epochs from one mouse on the REV day. w, wrong choices; c, correct choices. Post-dig activity on error trials that are followed by a correct choice are outlined. Right (top): Ca^2+^ traces for three error trials that are followed by a correct choice. Right (bottom): Ca^2+^ traces for three correct trials that follow a wrong choice. (**H**) Left: Example of dCA1 activity during post-digging epochs from one mouse on the REV day. Right (top): Ca2+ traces for three error trials that are followed by a correct choice. Right (bottom): Ca2+ traces for three correct trials that follow a wrong choice. (**I**) Post-dig Ca^2+^ activity for all trials from all mice on the REV day [Choice × Region, *F*(1,244) = 25.03, ***P < 0.0001; Choice, *F*(1,244) = 111.1, ***P < 0.0001; Region, *F*(1,244) = 2.74, *P* = 0.099; vCA1_wrong_ versus dCA1_wrong_, ***P < 0.0001]. (**J**) vCA1 post-dig activity correlated with activity during investigation on subsequent correct trials (*r* = 0.50, **P = 0.005). (**K**) Logistic regression shows a significant relationship between vCA1 post-dig activity and the probability of making a correct choice on the next trial (β_1_ = 0.18, |*Z*| = 3.58, ***P = 0.0003, OR = 1.194). (**L**) dCA1 post-dig activity did not correlate with activity during investigation on subsequent correct trials. (**M**) No relationship between dCA1 post-dig activity and probability of making a correct choice. *N* = 5 mice. Means ± SEM.

Next, we wanted to test whether the perseverative error signal in vCA1 is required for adaptation and whether the complementary roles of vCA1 and dCA1 in adaptation would extend also to nonspatial contingency changes. We therefore recorded both regions during a commonly used flexible odor/texture-based reward association task in which mice were presented with two bowls that were each marked with a different odor (O-1 and O-2) and filled with different textured digging media (T-A and T-B). In this task, mice first needed to learn that one of the odors (e.g., O-1) indicated that a reward was hidden in the respective bowl regardless of the spatial location or the digging medium texture (acquisition day, ACQ). Once mice reached a learning criterion of 80% correct choices across 10 consecutive trials, the reward-predicting feature was switched to the other odor (O-2) and mice needed to learn the reversed contingency (reversal learning day, REV). We then probed whether dCA1 and vCA1 would also be required for extradimensional set shifts (EDS), a form of cognitive flexibility that requires learning of a new feature-outcome contingency in a previously irrelevant feature dimension. For this purpose, the reward-predicting feature was shifted from the dimension of “odor” to the previously irrelevant dimension of “texture” (T-A or T-B) and mice needed to learn the new texture-reward contingency (Fig. 5, A and B).

Consistent with our findings in the Y maze and Barnes maze, dCA1 activity increased on the ACQ day during 3-s time periods when mice started investigating the two bowls before choosing to dig in the correct, rewarded bowl, but not when mice investigated the two bowls on the REV and EDS days (Fig. 5, C to F, red line). In contrast, vCA1 did not respond during bowl investigation on the initial ACQ day but increased on the REV and EDS days (Fig. 5, C to F, green line). No significant changes were observed when mice explored the two bowls before choosing to dig in the incorrect, nonrewarded bowl each day (fig. S9), indicating that hippocampus activity is associated with correct choice behavior also following nonspatial reversals and extradimensional shifts.

We then asked whether we would find a perseverative error signal also in this nonspatial reversal task. We thus analyzed Ca^2+^ signals following the termination of digging behavior in the incorrect bowl when mice failed to obtain reward on the REV day. Neural activity was generally higher after digging in the incorrect bowl than after digging in the correct bowl, and this difference in neural activity following incorrect versus correct choices was larger in vCA1 than in dCA1 (Fig. 5, G to I, and fig. S10A), suggesting a stronger role for vCA1 in processing perseverative errors than in dCA1. We then investigated whether perseverative error signals are related to the increased vCA1 activity that we observed during correct-choice behavior on the REV and EDS days (shown in Fig. 5C). We applied a linear regression model to examine the relationship between neural activity during the post-dig period after an error and neural activity during the periods when mice investigated the two bowls on subsequent correct-choice trials on the REV day. In vCA1, post-dig error activity significantly correlated with activity during investigation periods on subsequent correct trials (Fig. 5J and fig. S10B), indicating that perseverative error signals are associated with vCA1 activity during subsequent correct choices.

We then used logistic regression to test whether the post-dig error signal is also associated with subsequent correct choice behavior. We analyzed post-dig Ca^2+^ responses to correct and incorrect choices that immediately preceded behavioral transition points, that is, specifically on trials before mice selected a bowl that was different from the one chosen in the previous trial. We found a significant relationship between vCA1 post-dig neural activity and the probability of making a correct choice on the next trial with an odds ratio (OR) of 1.194, indicating that a one-unit increase in neural activity following a previous choice increases the odds of a subsequent correct choice by ~19.4%. Post-dig neural activity in vCA1 thus significantly predicted the probability of a subsequent correct choice (Fig. 5K and fig. S12B).

In dCA1, we did not find an association between post-dig neural activity and neural activity during subsequent correct choices (Fig. 5L) or between post-dig neural activity and the probability of a subsequent correct choice (Fig. 5M and fig. S12B). On the EDS day, we found a similar, albeit weaker, association between vCA1 post-dig neural activity and neural activity during subsequent correct choices and between post-dig neural activity and the probability of making a correct choice (OR = 1.093) (figs. S11, M and N, and S12C). No associations between post-dig neural activity and subsequent correct-choice neural activity or between post-dig neural activity and the probability of a subsequent correct choice were found on the ACQ day for either vCA1 or dCA1 (figs. S11, A to H, and S12A). A lack of an association between post-dig neural activity and subsequent choice behavior on the ACQ day supports the notion that this association is specific to perseverative errors but not to general learning errors.

**Fig. 6.**
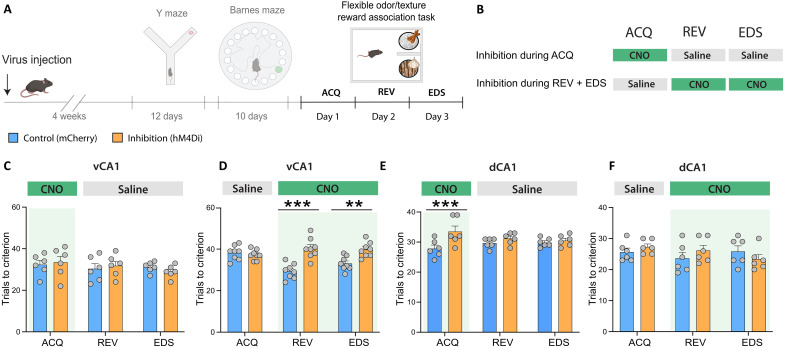
dCA1 and vCA1 inhibition effects on nonspatial associative learning, reversal learning, and extradimensional set shifting. (**A**) Experimental timeline. (**B**) Experimental design for CNO injections to inhibit vCA1 or dCA1 either during acquisition (ACQ) or during reversal (REV) and extradimensional set shifting (EDS). (**C**) No effect of vCA1 inhibition during ACQ on learning performance [Inhibition × Time, *F*(2,10) = 0.7184, *P* = 0.51; Inhibition, *F*(1,5) = 0.01, *P* = 0.92; Time, *F*(2,10) = 0.51, *P* = 0.5774]. (**D**) vCA1 inhibition during REV and EDS delayed learning after each contingency change (two-way ANOVA; Inhibition × Time, *F*(2,14) = 10.43, **P = 0.001, Time, *F*(2,14) = 2.666, *P* = 0.104; Inhibition, *F*(1,7) = 32.25, ***P = 0.0008; REV: ***P < 0.0001, EDS: **P = 0.003]. (**E**) dCA1 inhibition during ACQ delayed learning on the ACQ day [Inhibition × Time, *F*(2,10) = 5.435, *P = 0.025; Inhibition, *F*(1,5) = 16.61, **P = 0.009; Time, *F*(2,10) = 0.070, *P* = 0.932; ACQ: ***P = 0.0006]. (**F**) dCA1 inhibition during REV and EDS days did not affect learning after each contingency change [Inhibition × Time, *F*(2,20) = 1.57, *P* = 0.2329; Inhibition, *F*(1,10) = −0.1922, *P* = 0.67; Time, *F*(1.556,15.56) = 0.917, *P* = 0.397]. Means ± SEM.

Last, we inhibited vCA1 or dCA1 to test their functional roles in nonspatial REV and EDS in the flexible odor/texture-based reward association task (Fig. 6, A and B). In line with the observed neural activity signals in Fig. 5C, vCA1 inhibition during initial acquisition did not affect the number of trials mice needed to reach the learning criterion on the ACQ day (Fig. 6C), whereas inhibition on the REV and EDS days increased the number of trials needed to reach criterion (Fig. 6D). In contrast, dCA1 inhibition during initial rule acquisition delayed learning on the ACQ day (Fig. 6E) without affecting learning performance on the REV and EDS days (Fig. 6F).

Together, these results indicate that vCA1’s role in flexible behavior adaptation is to signal a perseverative error that facilitates subsequent adaptation of behavior following a contingency change, irrespective of whether the change occurs within or across feature dimensions. In contrast, dCA1 is required for learning regardless of whether the learning is related to spatial or nonspatial (e.g., odor) information.

## DISCUSSION

Identifying neural circuit components that contribute to cognitive flexibility will be important to improve our understanding of how the brain adapts to change. Our results across three tasks demonstrate specialized but complementary contributions of the dorsal and ventral hippocampus to flexible behavior adaptation. We highlight a previously unknown role for vCA1 in detecting perseverative errors, which we find to be required for adapting behavior to contingency changes, whereas dCA1 consolidates learned contingencies regardless of whether the consolidation occurs before or after a rule change. Our findings have particular relevance for understanding the biological basis of psychiatric or neurological disorders that are characterized by heightened perseveration or habitual behavior resulting from impaired cognitive flexibility.

Previous works have highlighted functional differences between the dorsal and ventral subregions of the hippocampus. These studies identified a predominant role for the dorsal hippocampus in spatial navigation and contextual memory (*33*, *34*) and a role for the ventral hippocampus in affective functions, such as stress response regulation and anxiety-like behavior (*19*, *25*, *26*, *35*–*37*). In addition to regulating affective states, the ventral hippocampus has been implicated in associative learning (*7*), response inhibition (*38*), goal-directed behavior (*39*, *40*), and goal value updating (*41*)—cognitive functions that are needed to efficiently adapt behavior (*39*).

To better understand how vCA1 facilitates adaptation to a new contingency, we determined neural Ca^2+^ responses during task-relevant events and behavior strategies, which revealed a previously unknown role for vCA1 in guiding behavior following the detection of perseverative errors. In the Y maze and Barnes maze tasks, vCA1 activity increased predominantly during lose-shift behaviors when mice adapted their responses upon detecting a mismatch between an expected and an actual outcome, and neural activity in vCA1 correlated with the number of times mice used lose-shift strategies. At the functional level, vCA1 inhibition increased the probability of repeating a previous incorrect choice and of changing behavior after a previous correct choice in the early phases of reversal learning. These behavior changes under conditions of vCA1 inhibition suggest a reduced ability to update behavior in response to a mismatch detection when vCA1 is taken offline. In the Barnes maze, vCA1 activity responded specifically to perseverative errors, but not to new learning errors or to correct choices, and inhibition increased the number of perseverative errors after changing the escape box location. vCA1 may thus facilitate cognitive flexibility by suppressing previously learned, no longer relevant behaviors during the early phases after a contingency change, whereas dCA1 is required for sustained learning of a contingency before and after a rule change. Such a role for vCA1 in suppressing perseverative responses is supported by previous lesion studies in rodents (*42*, *43*) and in line with a reported role for the ventral hippocampus in response inhibition (*44*), inhibitory control (*38*), and latent inhibition (*45*). Moreover, a recent work has shown that vCA1 forms abstract, nonspatial representations of latent contextual states that enable flexible decision-making and hidden state inference, further supporting its role in guiding behavior based on context-dependent expectations (*46*). A prior work has also shown that mice continue using previously learned behaviors when vCA1 is inhibited, suggesting that vCA1 may be required to terminate or update behaviors when they are no longer effective (*39*). Increased perseveration following vCA1 inhibition, as observed in our study, may thus lead to behavioral rigidity and cause the animal to persist in previously relevant behaviors despite new negative feedback, indicating that the prior outcome is now incorrect.

Human fMRI (functional magnetic resonance imaging) studies have found that the hippocampus responds to unexpected events (*47*) and that CA1 registers mismatches between expected and actual events akin to a prediction error (*48*–*51*). Such prediction error signals were found to be stronger in the human anterior hippocampus where they were associated with memory updating (*47*). In our rodent experiments, we find that the detection of a perseverative error triggers vCA1 activity and subsequently facilitates adaptation to a new contingency. For example, in the flexible odor/texture-based reward association task, perseverative error signals in vCA1 positively correlated with neural activity during subsequent correct choice trials. This association appears to be functionally important for adapting to the new rule as vCA1 perseverative error signals predicted correct choice behavior after a contingency change and vCA1 inhibition delayed reversal learning and extradimensional set shifting. These data, together with similar findings in the Y maze and Barnes maze task, support the notion that vCA1 detects a mismatch between expected and actual outcomes, which keeps vCA1 engaged until the perseverative response is terminated and learning of the new contingency begins. The mechanisms responsible for engaging vCA1 during these early stages of adaptation are, however, unknown. Considering that prediction errors have been closely linked with dopaminergic neuromodulation (*52*), it is possible that dopaminergic projections to vCA1 trigger neural activity changes after perseverative errors. These projections may originate from dopaminergic neurons in the ventral tegmental area or locus coeruleus, which both signal prediction errors and salience information to the hippocampus (*53*–*55*).

Our data indicate that, following a vCA1-dependent adaptation phase, dCA1 is required for the continued learning and consolidation of the new contingency. In the Y maze task, dCA1 activity is recruited during the later stages of reversal learning when vCA1 activity declines. dCA1 inhibition delayed learning performance and increased the probability of repeating a previous incorrect choice and of changing behavior after a previous correct choice during the later stages of reversal learning when vCA1 was no longer functionally required. This mechanistic role of dCA1 in memory consolidation appears to be independent on whether learning occurs before or after a rule change, as we find similar neural activity signals and behavioral effects of dCA1 inhibition during the initial rule acquisition. It is worth noting, however, that although we see a consistent rise in dCA1 activity during initial rule acquisition across all three tasks, dCA1 activity did not increase after the change in the escape hole location in the Barnes maze. dCA1 inhibition did, however, delay new learning after the rule change in the Barnes maze, as shown by reduced direct spatial search strategies and increased errors, latency, and distance traveled to find the new escape hole location. Although we recorded dCA1 on the first and last day after the change in the escape hole location (R1 and R5), it is possible that an increase in dCA1 activity could have been detected on the intermediate days after the rule change (R2 to R4), on which we did not collect Ca^2+^ activity data. A lack of a detectable increase in dCA1 activity after the rule change could also reflect differences in task demands or spatial memory processes between the Barnes maze and the Y maze. The Barnes maze relies heavily on global spatial cues and allocentric navigation, which are typically encoded by dCA1 during the initial learning phase, as we also see in our data. Once the contingency changes in the same spatial context, dCA1 may rely more on previously formed spatial representations rather than generating new representations to learn the new contingency. This is supported by our finding that dCA1 inhibition during the initial acquisition did reduce goal-directed spatial search strategies not only during acquisition but also when mice had to find the new escape hole location after the rule change without further inhibition. Mice with dCA1 inhibition during acquisition may therefore be unable to encode the spatial context necessary for using allocentric search strategies. Impairments in learning the spatial cues could subsequently hinder the ability to learn the new contingency even in the absence of further inhibition because the spatial configuration of the task still needs to be learned.

It is noteworthy that we found similar roles of dCA1 and vCA1 across three different tasks that probe complementary aspects of cognitive flexibility. This consistency underscores the generalizability of dCA1 and vCA1 functions in adaptation, which extend across reward-based and avoidance-based learning, egocentric and allocentric learning, and spatial and nonspatial learning. The dorsal hippocampus has predominantly been implicated in spatial learning based on the presence of defined place fields in dCA1 (*56*–*58*). In our experiments, dCA1 mediated associative learning in both spatial and nonspatial contexts, consistent with a previous study showing that dCA1 encodes odors and participates in odor-outcome learning (*7*). Moreover, in addition to facilitating reversal learning in both spatial and nonspatial contexts, vCA1 was required for adapting to a contingency change regardless of whether the change occurred within or across feature dimensions. Previous studies have shown that different cortical regions are required for reversal learning within one feature dimension as compared to extradimensional set shifting, in which rule changes occur across different feature dimensions. These prior studies have predominantly implicated the rodent orbitofrontal cortex (OFC) in reversal learning and the medial prefrontal cortex (mPFC) in set shifting. Some of our findings are reminiscent of studies that have reported similar findings in the OFC. OFC neurons have been shown to respond to rule changes (*59*) and to signal a perseverative error (*60*), and OFC responses following incorrect choices correlate with subsequent correct choice behavior (*61*). OFC lesions also impair reversal learning performance and increase the probability of repeating previous incorrect choices (*62*–*65*). These functional similarities between OFC and vCA1 in reversal learning may suggest that they are both elements of a wider neural circuitry implicated in flexible behavior adaptation. Considering that we find vCA1 to be implicated similarly in reversal learning and set shifting, it is likely that vCA1 projections to different cortical areas may be involved in controlling reversal learning versus extradimensional shifts. Future studies aimed at identifying which vCA1 downstream projections regulate these different aspects of cognitive flexibility will be important to unravel the complex neural circuitry underlying adaptive behavior.

Although we define vCA1 as the ventral half of CA1, our recording sites and virus-mediated inhibition spans areas of CA1 that include parts of what can be considered both ventral and intermediate CA1. It is also possible that, in our recording experiments, spread of GCamp6f virus to the DG may have led to some granule cells contributing Ca^2+^ signals to our recording data. However, in the few mice in which virus spread to the DG, the DG was >300 μm away from the fiber tip and the relative contribution of a such distal DG signal compared to the more proximal and more strongly labeled CA1 pyramidal cells should be considered minor and not confound our data. It is important to note that hippocampal CA1 is a highly heterogeneous structure both at the cellular and at the functional level. Fiber photometry recordings, as used in our study, can only detect changes in bulk Ca^2+^ fluorescence from a population of cells, which may obscure important differences in cell-specific activity patterns. Follow-up studies should thus be conducted using single-cell imaging or electrophysiological recording techniques to further elucidate the potentially complex cellular activity patterns underlying vCA1-dependent flexible adaptation of goal-directed behavior.

Perseveration resulting from deficits in cognitive flexibility increases rumination and habitual behavior, which are common symptoms of debilitating mental illnesses, such as major depression, generalized anxiety, post-traumatic stress disorder, or obsessive-compulsive disorder (*1*–*6*). Hippocampal dysfunction has repeatedly been implicated in the pathogenesis of many of these conditions, and impaired hippocampal regulation of cognitive flexibility may thus represent a key pathogenic mechanism underlying psychiatric disorders. Decoding the intricate regulation of cognitive flexibility by the hippocampus will thus be crucial for building a better understanding of the neurobiology underlying adaptive behavior and provide a foundation for developing more advanced targeted interventions to treat deficits in cognitive flexibility and associated brain disorders.

## MATERIALS AND METHODS

### Mice

Procedures were conducted in accordance with the US National Institutes of Health (NIH) Guide for the Care and Use of Laboratory Animals and approved by the New York State Psychiatric Institute (NYSPI) Institutional Animal Care and Use Committee (IACUC), approval no. NYSPI-1594. Male C57BL/6J wild-type mice were purchased from the Jackson Laboratory and group housed three to five per cage with ad libitum access to food and water on a 12-hour/12-hour light/dark cycle. All behavioral testing was conducted during the light period. Recording mice were housed three to five per cage before optical probe implantation surgery and single housed after surgery. For inhibition experiments, separate cohorts of mice were tested in behavior tasks for experiments with dCA1 or vCA1 inhibition and for experiments with inhibition during acquisition or reversal/rule change periods (four separate cohorts in total). Control and experimental (inhibited) mice were always tested together within the same cohort and on the same days.

### Viral constructs

AAV9.Syn.GCaMP6f.WPRE.SV40, AAV9-hSyn-hM4D(Gi)-mCherry, and AAV9-hSyn-mCherry were purchased from Addgene at a titer of 3 × 10^12^ to 4 × 10^12^ vg/ml. All viruses were diluted to a titer of 1.3 × 10^13^ vg/ml for injections.

### Stereotactic surgeries

For all surgical procedures, mice were anesthetized with 1 to 3% isoflurane at an oxygen flow rate of 1 liter/min and positioned in a stereotaxic frame (David Kopf Instruments) on a T/pump warm water recirculator (Stryker). An anesthesia plane was maintained at 1% isoflurane for the duration of surgery. Eyes were lubricated with opthalamic ointment. Fur was shaved, and the incision site was sterilized with betadine/ethanol swabs before surgery. Each mouse was monitored and received carprofen (subcutaneously, 5 mg/kg) for pain management for 3 days after surgery. Mice were housed for 5 weeks postoperatively before the start of behavior experiments to allow sufficient recovery from surgery and viral expression.

### Virus injections

AAV9-hSyn-hM4D(Gi)-mCherry and AAV9-hSyn-mCherry were bilaterally injected into either dCA1 or vCA1 using a 10-μl NanoFil syringe and a 33-gauge beveled needle (World Precision Instruments) at a constant speed of 100 nl/min. Craniotomies were bilaterally created using a dental drill, and virus was injected at the following coordinates: for dCA1, anterior-posterior (AP): −2.18 mm, medial-lateral (ML): 1.75 and 1.80 mm, and dorsal-ventral (DV): −1.50 mm, with 150 nl injected per site. For vCA1, AP: −3.16 mm, ML: 3.45 mm, and DV: −3.85, −3.50, and −3.25 mm, with 250-nl injection volume per site.

After each injection, the needle was left at the injection site inside the brain for an additional 5 min to aid diffusion from the needle tip and to prevent backflow. The needle was then slowly retracted and the scalp incision closed with Vetbond 3M.

### Fiber optic implantations

For in vivo Ca^2+^ imaging, mice underwent a single surgery in which AAV9.hSyn.GCaMP6f.WPRE.SV40 virus was injected unilaterally using a 10-μl NanoFil syringe and a 33-gauge beveled needle (World Precision Instruments) at a constant speed of 100 nl/min before implanting a fiber optic over the injection site. Fiber optics were 4.2 mm long with a 0.4-mm diameter and 0.37 numerical aperture (Doric). Three 1/16″ microscrews (Antrin Miniature Specialties Inc.) were inserted in evenly spaced locations around the implantation site. The fiber optic was lowered in 0.1-mm DV steps and then fixed to the skull with dental cement (Metabond). Viral injection coordinates were as follows: dCA1: −2.18 mm AP, ±1.75 and ±1.80 mm ML, and −1.50 mm DV, with 150 nl injected per site; vCA1: AP −3.16 mm, ML ±3.45 mm, and DV −3.85, −3.50, and −3.25 mm, with 250 nl injected per site (from the skull). Fiber optic placement coordinates were as follows: dCA1: −2.18 mm AP, ±1.70 mm ML, and −1.40 mm DV; vCA1: −3.16 mm AP, ±3.40 mm ML, and −3.50 mm DV (from the skull). Imaging experiments commenced 5 weeks following virus injection and fiber optic implantation.

### Behavioral testing

#### Y maze

Mice were food restricted to 85% body weight. Following food restriction, mice were habituated to a Y maze chamber and exposed to one trial in which a reward (sweet cereal powder) was in the left arm, followed by a second trial in which a reward was presented in the right arm. Mice were allowed to explore the maze on each trial until they consumed the reward. Following habituation, the reward was consistently presented in one arm and mice were trained to find the reward location over a period of 6 consecutive days, six trials per day with 3-min intertrial intervals (acquisition days 1 to 6). On each trial, mice were placed in the start arm and allowed to choose between the two choice arms. When the correct arm was chosen, mice were allowed to consume the reward. Wrong arm choices were followed by a 60-s time-out in the wrong arm during which the arm entrance was closed off by the experimenter. Arm choice was defined as all four paws entering an arm. During the reversal phase (days 7 to 12), the reward location was switched to the previously nonrewarded arm. Reward locations were always counterbalanced between mice in each group.

#### Barnes maze

Mice were habituated to a Barnes maze (Ø: 92 cm, 20 holes, 95 cm high; MazeEngineers) with spatial cues around the testing room for 10 to 15 min. The day after the habituation period, mice were allowed to explore the platform to find the escape hole (19 cm by 10 cm by 3 cm) location for 5 consecutive days. Each day consisted of 4x 3-min trials separated by 10-min intertrial intervals (acquisition days 1 to 5). Following the last acquisition day, the escape hole location was rotated by 135° and mice were tested for 5 additional days, each of which consisted of 4x 3-min trials per day, separated by 10- to 15-min intertrial intervals (reversal days 6 to 10). Ethovision XT (Noldus) was used to record all trials on each day and to quantify the # of head dips into incorrect holes before entering the target (# new learning errors), # of head dips into the previous target hole before reaching the new target after reversal (# perseverative errors), and latency to enter the escape hole. To quantify search strategy use (direct, local, serial, and global), individual trajectories for each mouse on each trial were analyzed using Ethovision XT and each trajectory was manually categorized into one of the four search strategies.

#### Flexible odor/texture-based reward association task

Mice were food restricted to 85% body weight, habituated to a 60 cm by 30 cm by 19 cm test chamber, and trained to dig in two baited bowls for 2 days. On the initial acquisition day (ACQ, day 1), one bowl was baited with a quarter of a sweet cereal and marked with cinnamon odor. The unbaited bowl was marked with garlic odor. Each bowl contained one of two differently textured digging media (wood chips and paper chips). Digging medium was varied across trials and did not predict reward. Mice were considered to have learned the odor-reward association after eight correct choices over the course of 10 consecutive trials. On the rule reversal day (REV, day 2), the previously unrewarded odor predicted reward, and mice were tested until they reached the criterion of 8 correct choices of 10 consecutive trials. On the extradimensional set shifting day (EDS, day 3), one of the previously irrelevant digging media predicted the reward, and mice were tested until they reached the learning criterion.

### CNO delivery

All mice received CNO (5 mg/kg, intraperitoneally) either on all acquisition days or on all reversal days in Y maze, Barnes maze, and ACQ or REV and EDS days in the flexible odor/texture-based reward association task. CNO was injected 30 min before the first trial each day.

### Perfusions and sectioning

Following behavior assessment, mice were anesthetized with ketamine/xylazine [ketamine (100 mg/ml) and xylazine (20 mg/ml)] and transcardially perfused with 30 ml of cold saline, followed by 30 ml of ice-cold 4% paraformaldehyde in saline. Brains were dissected and postfixed overnight in 4% paraformaldehyde at 4°C. Brains were then cryoprotected in 30% sucrose (w/v) with 0.02% NaN_3_ (w/v) for 2 days at 4°C and subsequently frozen in optimum cutting temperature (OCT) compound (Tissue-Tek) and stored at −80°C until cryostat sectioning. Serial sections (40 μm) were cut through the entire hippocampus on a cryostat (Leica CM3050S) and stored in phosphate-buffered saline (PBS) with 0.02% NaN_3_ until further processing by immunohistochemistry.

### Immunohistochemistry

Immunohistochemistry was conducted according to our established protocols (*66*, *67*). To visualize mCherry fluorescence for confirmation of DREADD virus injection sites, free-floating brain sections containing the dorsal hippocampus and ventral hippocampus were washed in PBS + 0.3% Triton X-100 (PBST), incubated in 10% normal donkey serum (NDS) in PBST for 2 hours, and incubated overnight at 4°C with primary antibody in 10% NDS/PBST (rabbit–anti-mCherry, 1:500, ab167453, Abcam). The next day, sections were washed three times with 1x PBST for 10 min and incubated in secondary antibody (donkey anti-rabbit IgG Alexa Fluor 594, Jackson ImmunoResearch) in 10% NDS/PBST for 2 hours at room temperature, washed with 1xPBS twice for 10 min, and incubated with 1x PBS + 4′,6-diamidino-2-phenylindole (DAPI) (1:5000; Sigma-Aldrich) for nucleic acid staining for 10 min. Sections were mounted onto glass slides with AquaPolymount anti-fade mounting medium and imaged on a Leica fluorescence microscope.

### Fluorescence microscopy

To identify immunoreactive cells, images of dCA1 and vCA1 were taken using a Leica Thunder Tissue Imager (Leica DM6 B, Leica Microsystems Inc.) and excited at 405 nm (DAPI), 488 nm (GCamp6f), and 594 nm (mCherry). Sections were imaged with a Leica 20x dry objective (numerical aperture: 0.80; working distance: 0.4 mm), with a field of view (FOV) of 553.6 μm by 553.6 μm, a pixel size of 0.5 μm by 0.5 μm, and a speed of 400 Hz.

### Ca^2+^ signal acquisition and processing

Ca^2+^ signals for individual trials were acquired using a two-site RZ10X photometry system (TDT) to record GCaMP6f fluorescence simultaneously from dCA1 and vCA1. Two sets of 405 nm (isosbestic control) and 465 nm (GCaMP6f excitation) light-emitting diodes (LEDs) were used. The 405- and 465-nm LEDs were filtered through Doric X-port minicubes with band-pass filters (405 to 410 and 460 to 490 nm, respectively) to minimize off-target light excitation. Excitation and fluorescence collection were facilitated through patch cords connected to the implanted cannulas. The LED power output at the patch cord tips was set to 40 to 45 μW for 405 nm and 100 to 120 μW for 465 nm. Emitted fluorescence signals were filtered through minicubes (500- to 540-nm green fluorescent protein emission band) and detected by photodetectors. Data acquisition was performed using Synapse software at a sampling rate of 1017 Hz. Signals were sinusoidally modulated (210 Hz for 405 nm and 330 Hz for 465 nm) and subsequently demodulated and low-pass filtered at 3 Hz online.

The raw data obtained for each channel were later preprocessed and analyzed using custom-made Matlab scripts. First, data were downsampled 10x and the first 6 s were trimmed to remove light-on artifacts. Δ*F*/*F* was calculated as 100x(*F* − *F*0)/*F*0, where *F* represents the 465-nm fluorescence at each time point, and *F*0 was calculated by applying a least-squares linear fit (polyfit function with degree 1) to the 405-nm fluorescence signal to align it with the 465-nm signal. The Δ*F*/*F* was then detrended by removing a least-squares linear fit to remove potential slow drift and *z*-scored to normalize across animals and sessions.

### Z-scoring

For each session, the entire Δ*F*/*F* trace was *z*-scored by subtracting the session mean and dividing by the session SD to normalize session-to-session variability in recording conditions between mice and brain regions. All preprocessing steps were performed in MATLAB.

### Analysis of neural activity during defined behavior epochs

In the Y maze, the time during which mice traversed the transition zone was manually scored using BORIS software and defined as the 3-s period after which mice entered the triangular area preceding entry into any arm of the maze (corresponding to “choice” epochs). Entry was defined as both front paws passing the edge of the transition zone facing the start arm. Outcome-related epochs (reward versus no reward) were defined as the 3-s time period after mice finished consuming the reward on correct trials and as the 3-s time period after mice failed to find the reward after exploring the incorrect maze arm on incorrect trials.

In the Barnes maze, the following behavioral epochs were manually scored using BORIS: “Searching,” defined as periods when mice begin searching the maze. The beginning of each search epoch was aligned to the time when mice start exploring the maze, i.e., right at the beginning of placing the mice in the center of the maze and then each time mice travel back into the central area of the platform after exploring the holes around the perimeter. Neural activity was quantified over a period of 5 s following the onset of each search bout; “Investigating the target hole,” defined as head dips into the target hole containing the escape box; “Investigating any incorrect hole,” defined as head dips into any hole that did not contain the escape box; “Investigating previous hole,” defined as head dips into the hole that contained the escape box before the rule change. Neural activity was quantified over a period of 15 s following head deflections into a hole. This 15-s time window was chosen because it corresponds to the time period during which neural activity remained elevated following perseverative errors (“Investigating previous hole”; Fig. 3G).

In the flexible odor/texture-based reward association task, the behavioral epochs were defined as follows: “Investigating the correct bowl,” defined as the 3-s time period following the onset of correct bowl investigation (i.e., sniffing or searching around the bowl containing the reward) before starting to dig inside the bowl; “Investigating wrong bowl,” defined as the 3-s time period following the onset of wrong bowl investigation before starting to dig inside the bowl; “Post-dig correct,” defined as the start of the offset of digging in the baited bowl (i.e., following reward attainment); “Post-dig wrong,” defined as the start of the offset of digging in the unbaited bowl. As soon as the animal started digging in any of the two bowls, the entrance to that side of the chamber was blocked by the experimenter and mice remained in the blocked-off compartment for 30 s. “Post-dig correct” and “Post-dig wrong” epochs were defined as the entire period from digging offset to the end of the trial.

### Quantification of neural activity during defined behavior epochs

To quantify neural responses to specific behavioral events (e.g., transition zone entry, beginning of search epoch, and bowl investigation), processed Δ*F*/*F* traces were aligned to the onset of the event. To isolate event-related changes in Ca^2+^ fluorescence from pre-event activity, *z*-scored Δ*F*/*F* traces were baseline corrected by subtracting the mean signal from the −5- to 0-s period preceding the event onset. For group-level analyses of neural signals with fixed duration (e.g., 3 s following transition zone entry in the Y maze or 5 s following search onset in the Barnes maze), these event-aligned traces were first averaged across trials for each mouse. The resulting per-mouse means were then averaged across mice within each experimental group.

For trial-based analysis of epoch-related neural activity with varying length (“Post-dig correct” and “Post-dig wrong” epochs in Fig. 5I), we quantified the mean *z*-score Δ*F*/*F* signal over the entire epoch and divided the mean *z*-score by the respective epoch length.

### Quantification and statistical analysis

#### Statistical analysis of behavior

A state-space model and logistic regression curve fitting were independently used to examine mouse behavior during flexible choice training in the Y maze.

##### State-space model

We used a state-space model (Figs. 1C and 2, C to F) based on the approach described by Smith *et al.* (*32*), which provides a statistical framework for analyzing binary (correct/incorrect) behavioral data. This model estimates the probability of a correct response on a trial-by-trial basis while accounting for inherent variability in performance.

The model assumes an unobserved learning state *x_t_*, which evolves over time according to a simple random walk

$$x_{t\;}=\;x_{t-1}\;+\;w_{t}$$

where *w_t_* ∼ *N*(0, σ^2^) represents the Gaussian noise capturing trial-to-trial variability. The latent state *x_t_* is then transformed into an observable probability of a correct response using the following logistic function

$$p_{t}=1/[1+\text{exp}\left(-x_{t}\right)\text{]}$$

On each trial *t*, the observed outcome *y_t_* is binary (1: correct; 0: incorrect), with the likelihood defined as

$$P\left(y_{t}=1\right)=p_{t},P\left(y_{t}=0\right)=1-p_{t}$$

Model parameters were estimated using a maximum a posteriori (MAP) approach with a first-order Gaussian random walk prior. The priors were specified as follows: (i) Initial state prior: *x*_0_ ~ *N*(0, σ_0_^2^) where σ_0_^2^ = 1.0, indicating no prior bias in performance as animals had no prior knowledge of the Y maze task (i.e., 50% chance of correct response). During estimation, *x*_0_ was reestimated each expectation-maximization iteration using an empirical Bayes approach, which allows the model to learn the true starting performance level for each subject. (ii) Process noise variance σ^2^, which controls temporal smoothness of the trajectory, was initialized at 0.1 and then updated iteratively from the data using an empirical Bayes approach, so the model could adjust to the level of variability in each subject’s behavior. (iii) Convergence criteria: Absolute tolerance was set to 0.01 and relative tolerance to 0.001, with a maximum of 100 iterations, to ensure reliable and stable MAP estimation (as in Smith and Brown) (*68*).

To facilitate visualization, we applied a five-trial moving average to the estimated trial-by-trial probabilities, which reduces residual trial-to-trial fluctuations. This additional curve smoothing was only used for visualization and was not part of the model fitting process or statistical analysis. All modeling and visualization procedures were done in MATLAB.

##### Learning curve analysis using logistic functions

To quantify learning, we fitted a logistic regression curve to the binary behavioral data (correct: 1; incorrect: 0) for each mouse to model the probability of a correct response as a function of trial progression under the assumption that learning follows a sigmoidal trajectory over time (fig. S6). The logistic function was defined as follows

$$p_{t}=1/\left\{1+\text{exp}\left[-\left(\beta_{0\;}+\;\beta_{1}*t\right)\right]\right\}$$

where *p_t_* is the predicted probability of a correct response on trial *t*, β_0_ is the intercept, and β_1_ is the slope parameter reflecting the learning rate over trials.

Logistic curve fitting was performed in MATLAB using the glmfit function with a binomial distribution and logit link function. Predicted probabilities for each trial were obtained using glmval. Learning curve analysis was performed separately for two trial ranges: trials 1 to 36 (acquisition phase) and trials 36 to 72 (reversal phase). SEM values were calculated and used to visualize variability in the learning curves. To generate group-level learning curves, we averaged the predicted probabilities from each mouse and smoothed them using a moving average with a five-trial window. This smoothing was applied only for visualization purposes and was not part of the model fitting process.

##### Probability of choice strategies

To quantify choice strategies in the Y maze, we calculated the probability of staying or shifting choices following previous correct or incorrect trial outcomes as follows:

Probability of staying after an incorrect trial:

*P*(stay|lose) = (number of trials in which the mouse stayed with the same arm after an incorrect trial)/(number of trials where the previous trial was incorrect)

Probability of shifting after a correct trial:

*P*(shift|win) = (number of trials where the mouse shifted to the other arm after a correct trial)/(number of trials where the previous trial was correct)

Because, after any trial in the Y maze binary choice task, the mouse either stays with its previous choice or switches, the probabilities of staying and shifting after a given outcome are complementary [i.e., *P*(stay|outcome) + *P*(shift|outcome) = 1].

*P*(stay|lose) and *P*(shift|win) thus fully describe the strategy distribution following wins or losses.

All other behavioral data, including learning curves and strategy use in the Y maze; total errors, perseverative errors, latency, path length, and strategy use in the Barnes maze; and trials to criterions in the flexible odor/texture-based reward association task, were analyzed using repeated measures (RM) two-way analysis of variance (ANOVA) to assess inhibition (mCherry versus hM4Di) and time (days) effects. Tukey’s post hoc test was used to compare relevant groups when significant interactions were found.

#### Statistical analysis of neural activity data

To determine whether neural activity in dCA1 or vCA1 was elevated above the baseline on any given recording day in the Y maze and Barnes maze and in the flexible odor/texture-based reward association task, two-way RM ANOVA with Tukey’s post hoc test was used on *z*-scored Δ*F*/*F* signals for the respective behavior epochs (e.g., 3-s post–transition zone entry in the Y maze). dCA1 and vCA1 signals were each compared to “0.” To compare dCA1 and vCA1 Ca^2+^ activity signals with each other on specific days, paired *t* tests were used to compare *z*-scored Δ*F*/*F* signals for the respective time bins in each task. Neural activity for different strategy use on each day of Y maze testing was analyzed using one-way ANOVA and individual groups (strategies) were compared using Tukey’s post hoc test when a significant main effect was found. Pearson correlation was used to assess correlations between *z*-scored data and number of lose-shift trials on R1 in the Y maze and between post-dig Ca^2+^ activity *z*-scores and Ca^2+^ activity *z*-scores during investigation of the correct bowl on subsequent correct-choice trials in the flexible odor/texture-based reward association task. Logistic regression was used to determine the probability of a correct choice from post-dig Ca^2+^ activity *z*-scores on trials that preceded a switch to choosing to dig in a different bowl. Linear mixed-effects models with “mouse” as a random factor were used to account for cross-subject variability in trial-level group comparisons of Ca^2+^ activity *z*-scores. The following structure was used for the model: fixed effects: PrevOutcome (two levels: correct/incorrect), Region (two levels: vCA1/dCA1), PrevOutcome × Region interaction (to test whether the effect of previous outcome differs between regions, Intercept (baseline Ca^2+^ activity when all predictors are at their reference levels). Random effects: Mouse (random intercept for each subject to account for repeated measures). Model formula: Value ~ PrevOutcome*Region + (1|Mouse). Leave-one-out analyses (excluding data from one mouse at a time) were performed to assess the robustness of group effects. Data were confirmed to be normally distributed using the Shapiro-Wilk test. All statistical analyses were carried out using GraphPad Prism software (version 10.4.1) and MATLAB. Data were considered significant if *P* < 0.05.
