# Supplementary material for: Complementary roles of dorsal and ventral hippocampus in the flexible adaptation of goal-directed behavior
Source: Sci Adv. 2025 Nov 28;11(48):eadx7514. doi: 10.1126/sciadv.adx7514 (PMC12662218; doi:10.1126/sciadv.adx7514)
Supplement: Supplementary file 1 — Figs. S1 to S12 [file sciadv.adx7514_sm.pdf]

Science Advances
AAAS

Supplementary Materials for

Complementary roles of dorsal and ventral hippocampus in the flexible adaptation of goal-directed behavior

Maryam Hasantash *et al.*

Corresponding author: Christoph Anacker, ca2635@cumc.columbia.edu

Sci. Adv. **11**, eadx7514 (2025)
DOI: 10.1126/sciadv.adx7514

This PDF file includes:

Figs. S1 to S12

SUPPLEMENTARY FIGURES AND LEGENDS

Supplementary Figure 1

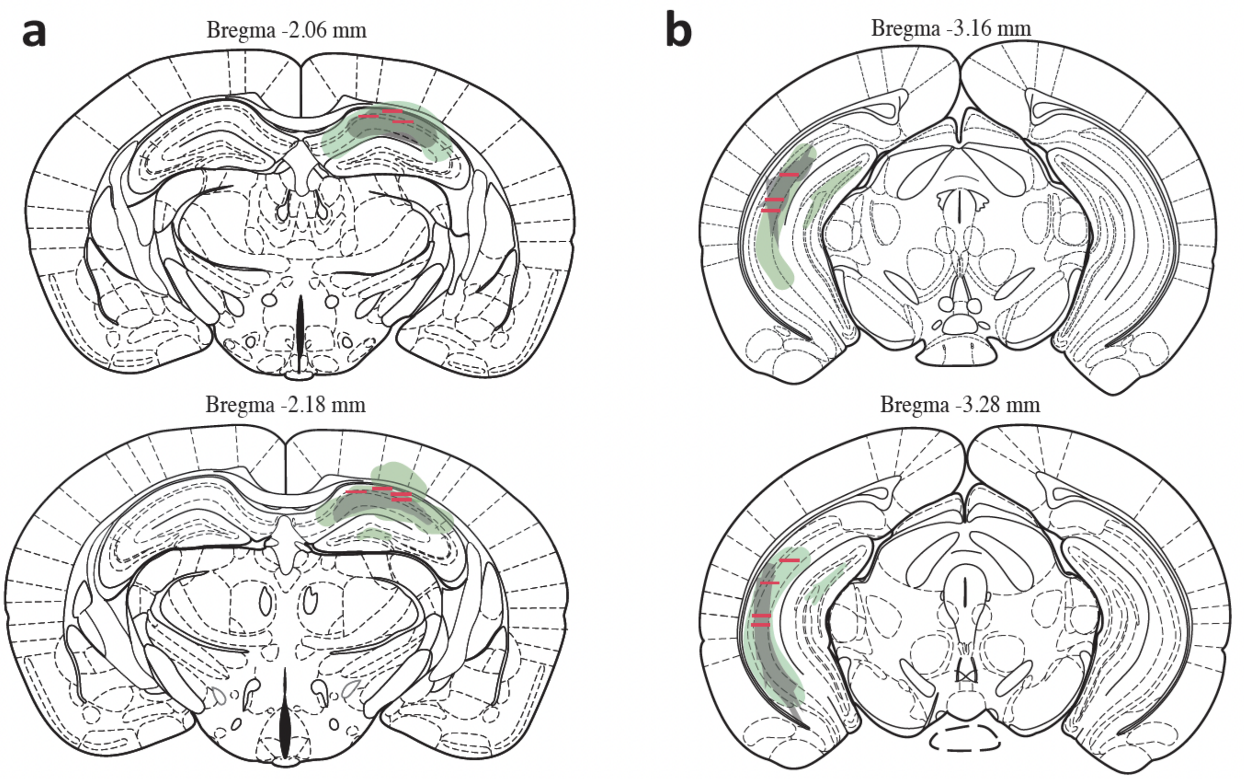

Supplementary Figure 1. Fiber placements in dCA1 and vCA1, related to Figure 1. a. Fiber placements in dCA1 **b.** Fiber placement in vCA1. Red lines indicate the tip of the lens. Grey shaded areas indicate minimum virus spread. Green shaded areas indicate maximum virus spread.

Supplementary Figure 2:

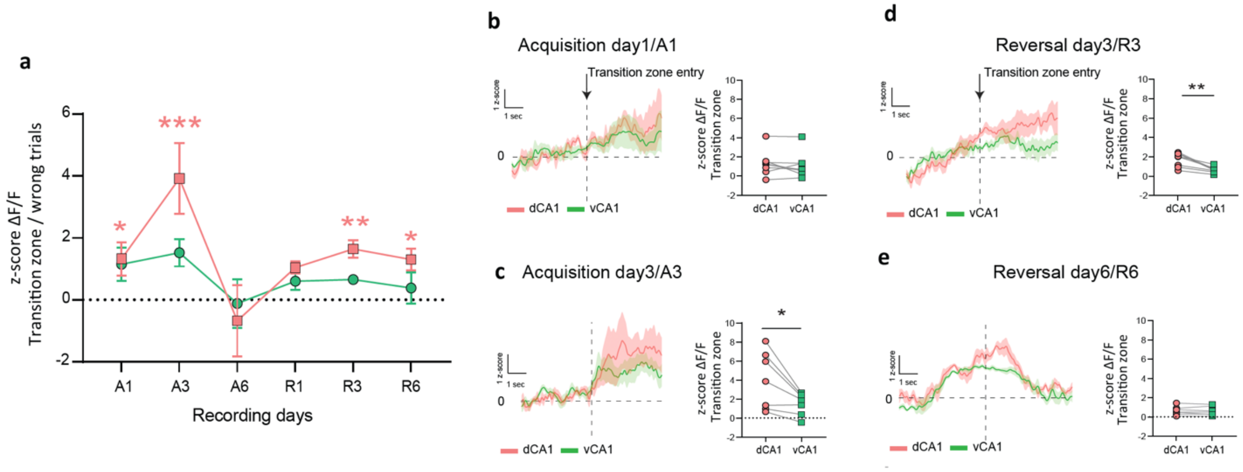

Supplementary Figure 2. Ca^{2+} activity in the transition zone during wrong trials in the Y Maze, related to Figure 1. a. Ca^{2+} activity in dCA1 and vCA1 during the 3-sec period upon entering the transition zone on wrong-choice trials on acquisition and reversal days. 2-Way RM ANOVA: Region × Time, $F(5,16)=4.3$, *P=0.01; Region, $F(1,6)=12.8$, *P=0.01; Time, $F(5,30)=3.4$, *P=0.02; $n_{dCA1}=7$ mice, $n_{vCA1}=7$ mice). Compared to baseline, dCA1 activity (red line) increased on A1 (*P=0.03), A3 (***P<0.0001), R3 (**P=0.008) and R6 (*P=0.046). **b–e.** Ca^{2+} signals aligned to transition zone entry on recording days with significantly elevated activity in either dCA1 or vCA1. Left: Average z-scored Ca^{2+} traces. Right: Average Ca^{2+} signal per mouse (z-scored ΔF/F over 3-sec period upon entering the transition zone). Paired t-test: A1 (P>0.05), A3 (*P=0.020), R3 (**P=0.002), R6 (P>0.05). Mean±S.E.M.

Supplementary Figure 3.

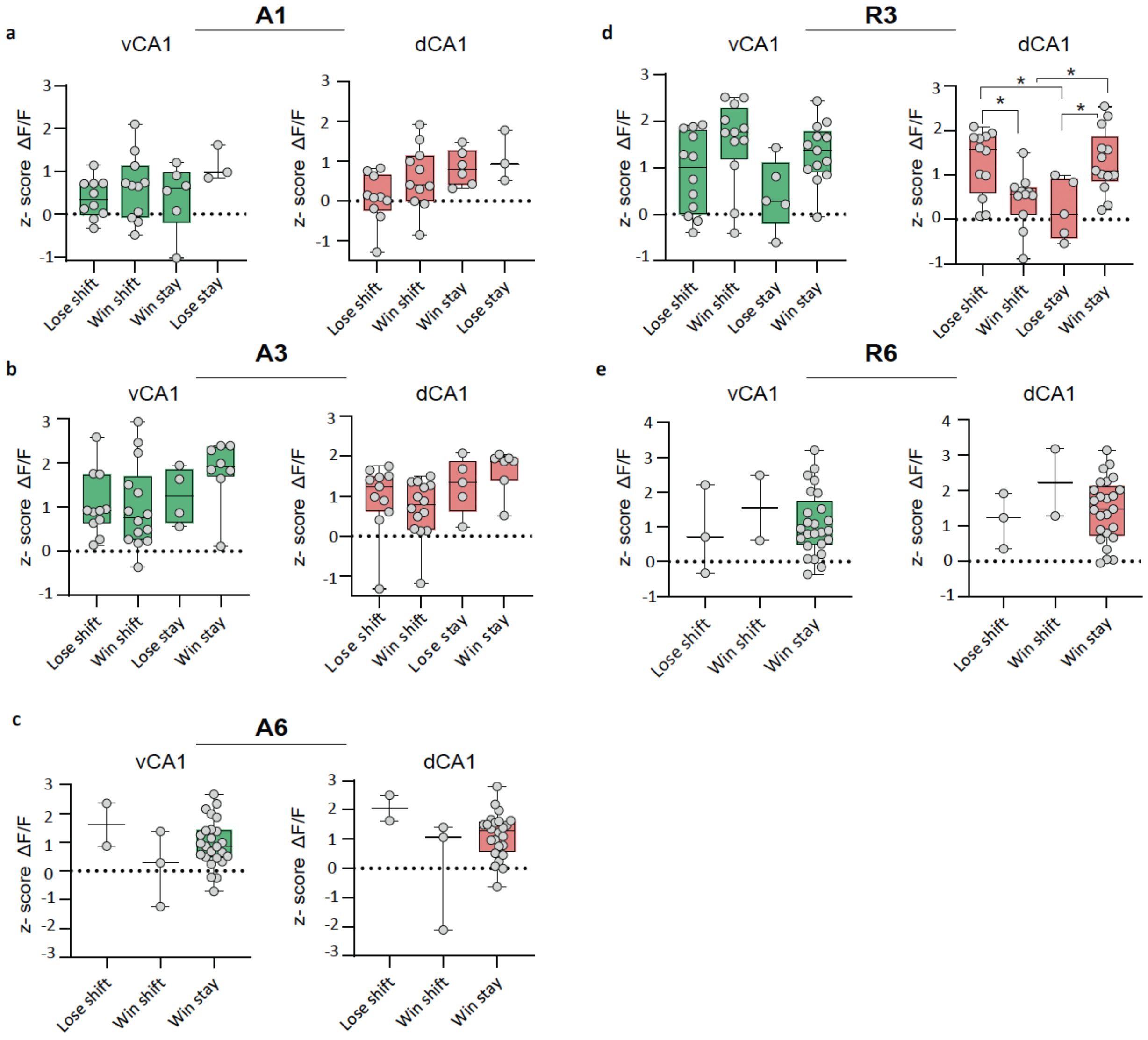

Supplementary Figure 3. Neural activity in dCA1 and vCA1 during strategy use in the Y Maze, related to Figure 1. a. No significant differences in average Ca^{2+} activity (z-scored ΔF/F) in the transition zone across trials with different strategy use on Acquisition Day 1 (A1) for vCA1 (One-way ANOVA, F(3,30)=1.23, P=0.31) or dCA1 (F(3,26)=2.73, P= 0.06). **b.** No significant differences in average Ca^{2+} activity (z-scored ΔF/F) across trials with different strategy use on Acquisition Day 3 (A3) for vCA1 (F(3,33)=1.84, P=0.158) or dCA1 (F(3,33)=2.88, P=0.05). **c**. No significant differences in average Ca^{2+} activity (z-scored ΔF/F) across trials with different strategy use on Acquisition Day 6 (A6) for vCA1 (F(2,27)=1.81, P=0.18) or dCA1 (F(2,26)=2.88, P=0.072). **d.** Average Ca^{2+} activity (z-scored ΔF/F) across trials with different strategy use on Reversal Day 3 (R3) (vCA1: F(3,38)=2.94, *P=0.045, all *post hoc* comparisons, P>0.05; dCA1: F(3,37)=5.81, **P=0.002, Tukey *post hoc* test: Lose-shift vs. Win-shift, *P=0.031, Lose-shift vs. Lose-stay, *P=0.033, Win-shift vs. Win-stay, *P=0.022, Lose-stay vs. Win-stay, *P=0.026). **e.** No significant differences in average Ca^{2+} activity (z-scored ΔF/F) across trials with different strategy use on Reversal Day 6 (R6) for vCA1 (F(2,27)=0.3, P=0.73) or dCA1 (F(2,27)=0.88, P=0.42). Mean±S.E.M.

Supplementary Figure 4.

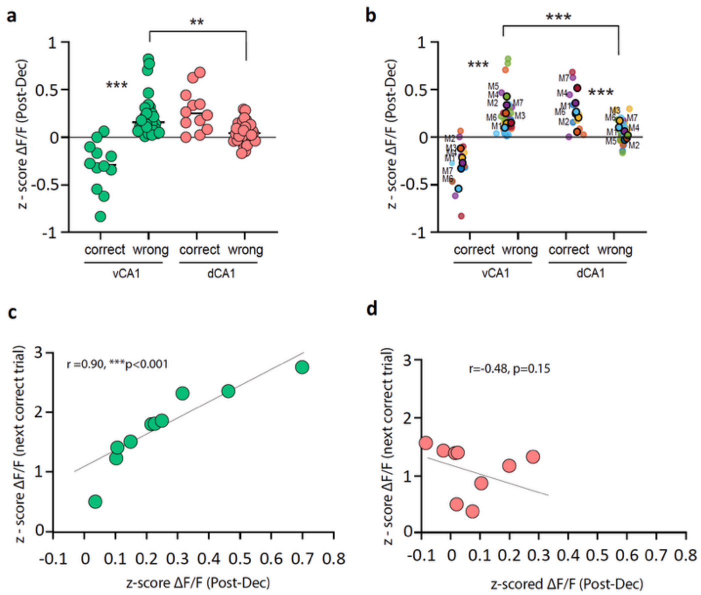

Supplementary Figure 4. Outcome-related activity in dCA1 and vCA1 in the Y Maze on reversal day R1, related to Figure 1. a. Ca^{2+} activity (z-scored ΔF/F) for all trials from all mice on the R1 day. Error-related activity during a 3-sec period after lack of reward attainment (wrong trials) was higher in vCA1 than dCA1 (Choice × Region, $F(1,84)=69.55$, ***$P<0.0001$; Choice, $F(1,84)=10.75$, **$P=0.0015$; Region, $F(1,84)=21.23$, ***$P<0.0001$; $vCA1_{correct\ choice}$ vs $vCA1_{wrong\ choice}$, ***$P<0.0001$; $vCA1_{wrong\ choice}$ vs $dCA1_{wrong\ choice}$, **$P=0.003$). **b.** Linear mixed-effects model (Choice × Region, β=0.753, ***$P<0.0001$; dCA1, β=-0.23, ***$P<0.0001$; vCA, β=0.525, ***$P<0.0001$; n=7 mice). Leave-one-out analysis, confirmed that these effects were not driven by any single mouse, ***$P<0.0001$ for interaction. N=7 mice **c.** vCA1 error signals positively correlated with activity in the transition zone on subsequent correct trials (r=0.90, ***$P<0.0001$). **d.** dCA1 error signals did not correlate with activity in the transition zone on subsequent correct trials (r=-0.48, P=0.15). Mean±S.E.M.

Supplementary Figure 5.

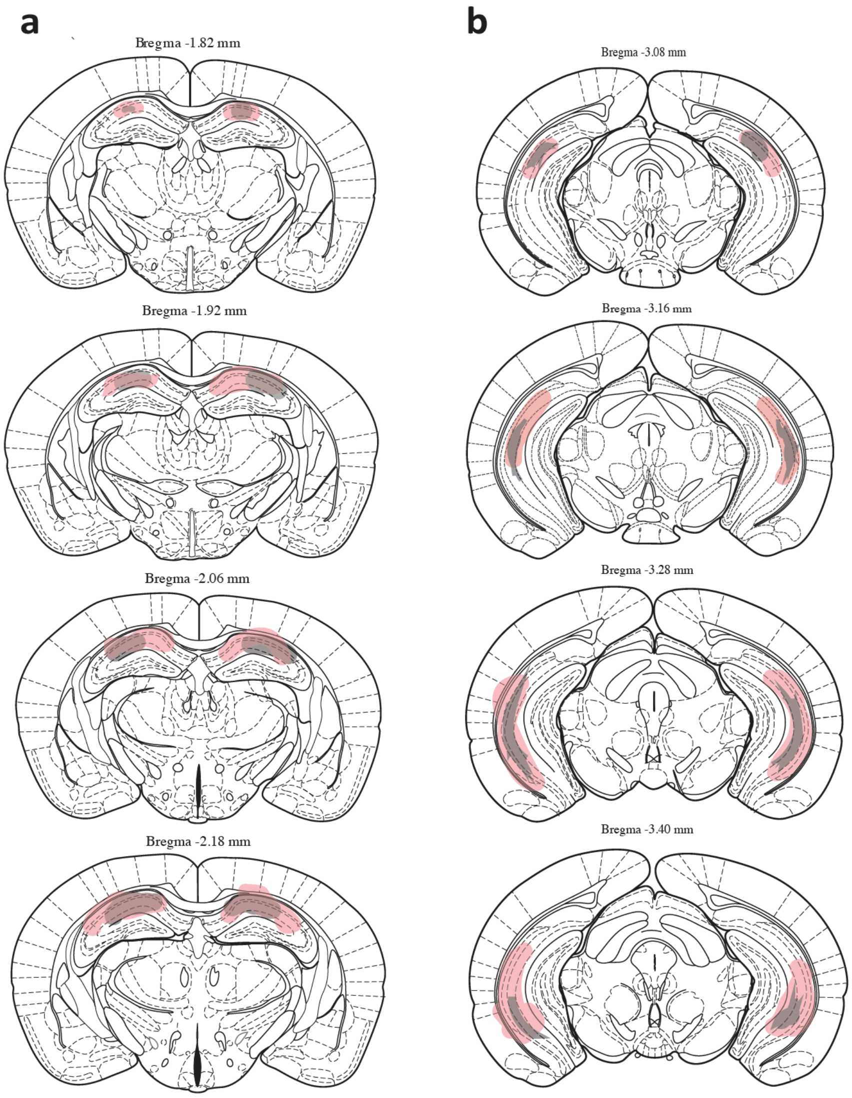

Supplementary Figure 5. hM_4D_i virus injections in dCA1 and vCA1, related to Figure 2. a. Virus spread in dCA1 **b.** Virus spread in vCA1. Grey shaded areas indicate minimum virus spread. Red shaded areas indicate maximum virus spread.

Supplementary Figure 6.

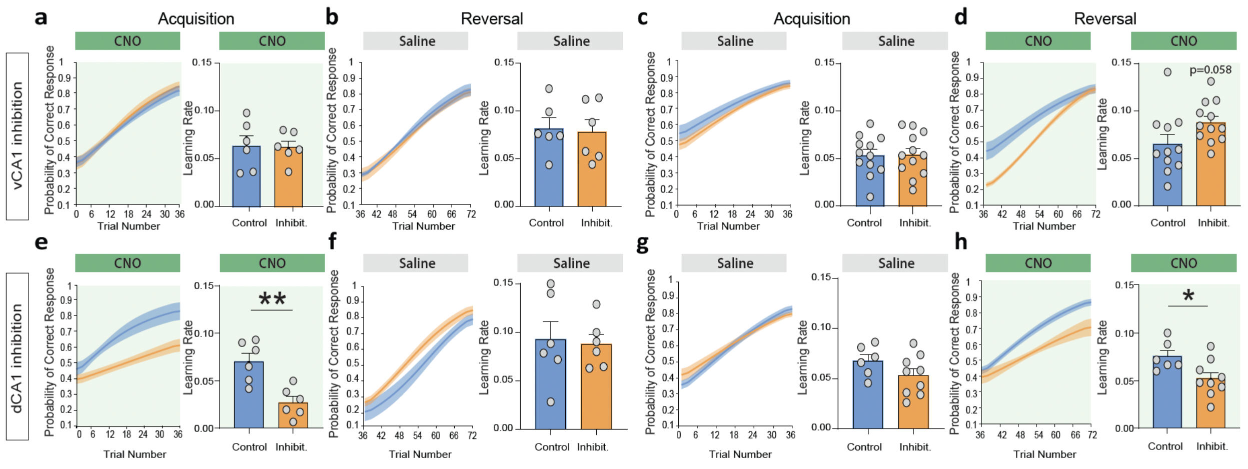

Supplementary Figure 6. Learning rate analysis indicates delayed reversal learning performance following vCA1 inhibition, related to Figure 2. Left panels: Learning curves (logistic curve fitting); Right panels: learning rate comparisons between control and inhibition groups. **a.** vCA1 inhibition during acquisition does not affect learning rate during acquisition (P=0.91) or **b**, during reversal (P=0.84). **c.** vCA1 inhibition during reversal does not affect learning rate during the preceding acquisition phase (P=0.92), but **d,** causes a lower probability of making a correct choice immediately after reversal, with a trend toward faster learning during the late reversal learning phase (P=0.058). **e.** dCA1 inhibition during acquisition decreases learning rate during acquisition (**P=0.002) but **f,** not during reversal (P=0.81). **g.** dCA1 inhibition during reversal does not affect learning rate during the preceding acquisition phase (P=0.16), but **h**, slows down reversal learning without affecting the probability of making a correct choice immediately following the reversal (*P=0.027). Mean±S.E.M.

Supplementary Figure 7.

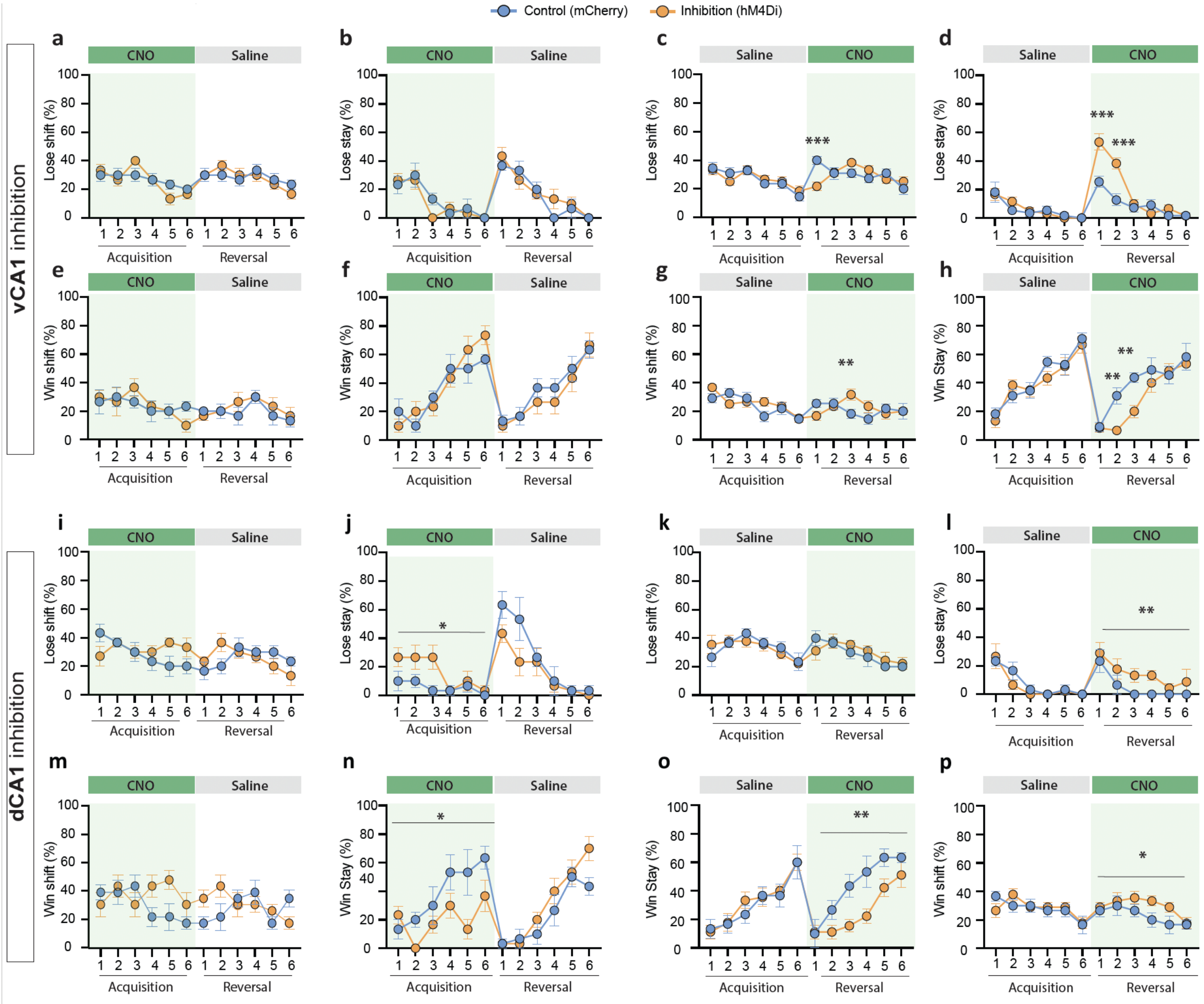

Supplementary Figure 7. Adaptive strategy use in the Y Maze, related to Figure 2. a-h. Effect of vCA1 inhibition by CNO in hM4Di-injected mice. **a.** vCA1 inhibition during acquisition did not affect lose shift strategy use (2-Way RM ANOVA, Inhibition × Time, F(5,50)=1.73, P=0.15; Inhibition, F(1,10)=0.05, P=0.83; Time, F(5,50)=7.05, ***P<0.0001; $n_{control}$=6, n_{hM4Di}=6). **b.** vCA1 inhibition during acquisition did not affect lose-stay strategy use (Inhibition × Time, F(5,50)=0.85, P=0.52; Inhibition, F(1,10)=0.62, P=0.45; Time, F(5,50)=12.41, ***P<0.0001; $n_{control}$=6, n_{hM4Di}=6). **c.** vCA1 inhibition after reversal decreased lose-shift strategy use immediately after reversal (Inhibition × Time, F(5,105)=5.79, ***P<0.0001; Inhibition, F(1,21)=0.05, P=0.82; Time, F(5,105)=3.996, **P=0.002; Tukey *post hoc* test, R1: ***P<0.0001; $n_{control}$=11, n_{hM4Di}=12). **d.** vCA1 inhibition during reversal increased lose-stay strategy use (Inhibition × Time, F(5,105)=8.35, ***P<0.0001; Inhibition, F(1,21)=25.76, ***P<0.0001; Time, F(5,105)=37.52, ***P<0.0001;

Tukey's *post hoc* test: R1, ***P<0.0001; R2, ***P<0.0001; $n_{control}$=11, n_{hM4Di}=12). **e.** vCA1 inhibition during acquisition did not affect win-shift strategy (Inhibition × Time, F(5,50)=0.87, P=0.509; Inhibition (F(1,10)=0.00, P>0.999; Time F(5,50)=1.90, P=0.11; $n_{control}$=6, n_{hM4Di}=6) or **f.** win-stay strategy (Two-Way RM ANOVA, Inhibition × Time, F(5,50)=1.37, P=0.25; Inhibition F(1,10)=0.43, P=0.52; Time F(5,50)=18.39, ***P<0.0001). **g.** vCA1 inhibition during reversal learning increased Win shift on day R3 (Inhibition × Time, F(5,105)=2.81, *P=0.02; Inhibition, F(1,21)=0.29, P=0.59; Time, F(5,105)=1.02, P=0.41; Tukey's *post hoc* test, R3: **P=0.009, $n_{control}$=11, n_{hM4Di}=12) and **h.** decreased Win-stay (Inhibition × Time, F(5,105) = 2.28, P = 0.05; Inhibition, F(1,21)=7.49, *P=0.012; Time: F(5,105)=22.21, ***P < 0.0001; Tukey's *post hoc* test, R2: **P=0.003, R3: **P=0.003; $n_{control}$=11, n_{hM4Di}=12). **i-p.** Effect of dCA1 inhibition by CNO in hM4Di-injected mice. **i.** Lose shift strategy use under dCA1 inhibition during acquisition (Inhibition × Time, F(5,50)=2.63, *P=0.035; Inhibition, F(1,10)=0.67, P=0.43; Time, F(5,50)=1.44, P=0.23; $n_{control}$=6, n_{hM4Di}=6). **j.** dCA1 inhibition during acquisition increased lose-stay strategy use (Inhibition × Time, F (5,50)=1.66, P=0.16; Inhibition, F(1, 10)=7.5, *P=0.02; Time, F(5,50)=4.1, **P=0.0037; $n_{control}$=6, n_{hM4Di}=6). **k.** No effect of dCA1 inhibition during reversal on lose-shift strategy use (Inhibition × Time, F(5,65)=0.66, P=0.66; Inhibition, F(1,13)=0.2, P=0.66; Time, F(5,65)=4.27, **P=0.002; $n_{control}$=6, n_{hM4Di}=9). **l.** dCA1 inhibition during reversal increases lose-stay strategy use (Inhibition × Time, F(5,65)=0.196, P=0.96; Inhibition, F(1,13)=11.93, **P=0.004; Time, F(5,65)=4.034, **P=0.003; $n_{control}$=6, n_{hM4Di}=9). **m.** dCA1 inhibition during acquisition did not affect Win shift strategy (Inhibition × Time, F(5,50)=2.09, P=0.08; Inhibition F(1,10)=2.66, P=0.13; Time F(5,50)=1.100, P=0.37; $n_{control}$=6, n_{hM4Di}=6) but **n,** decreased Win-stay strategy use during acquisition (Inhibition × Time, F(5,50)=1.93, P=0.11; Inhibition F(1,10)=6.81, *P=0.02; Time F(5,50)=6.24, **P=0.001). **o.** dCA1 inhibition during reversal learning decreased Win-stay strategy use (Inhibition × Time, F(5,65)=1.46, P=0.22; Inhibition F(1,13)=18.49, **P=0.009; Time F(5,65)=14.59, ***P<0.0001; $n_{control}$=6, n_{hM4D}=9), and **p,** increased Win-shift strategy use (Inhibition × Time, F(5,65)=0.66, P=0.65; Inhibition, F(1,13)=5.86, *P=0.03; Time, F(5,65)=2.77, *P=0.048; $n_{control}$=6, n_{hM4D}=9). Mean±S.E.M.

Supplementary Figure 8.

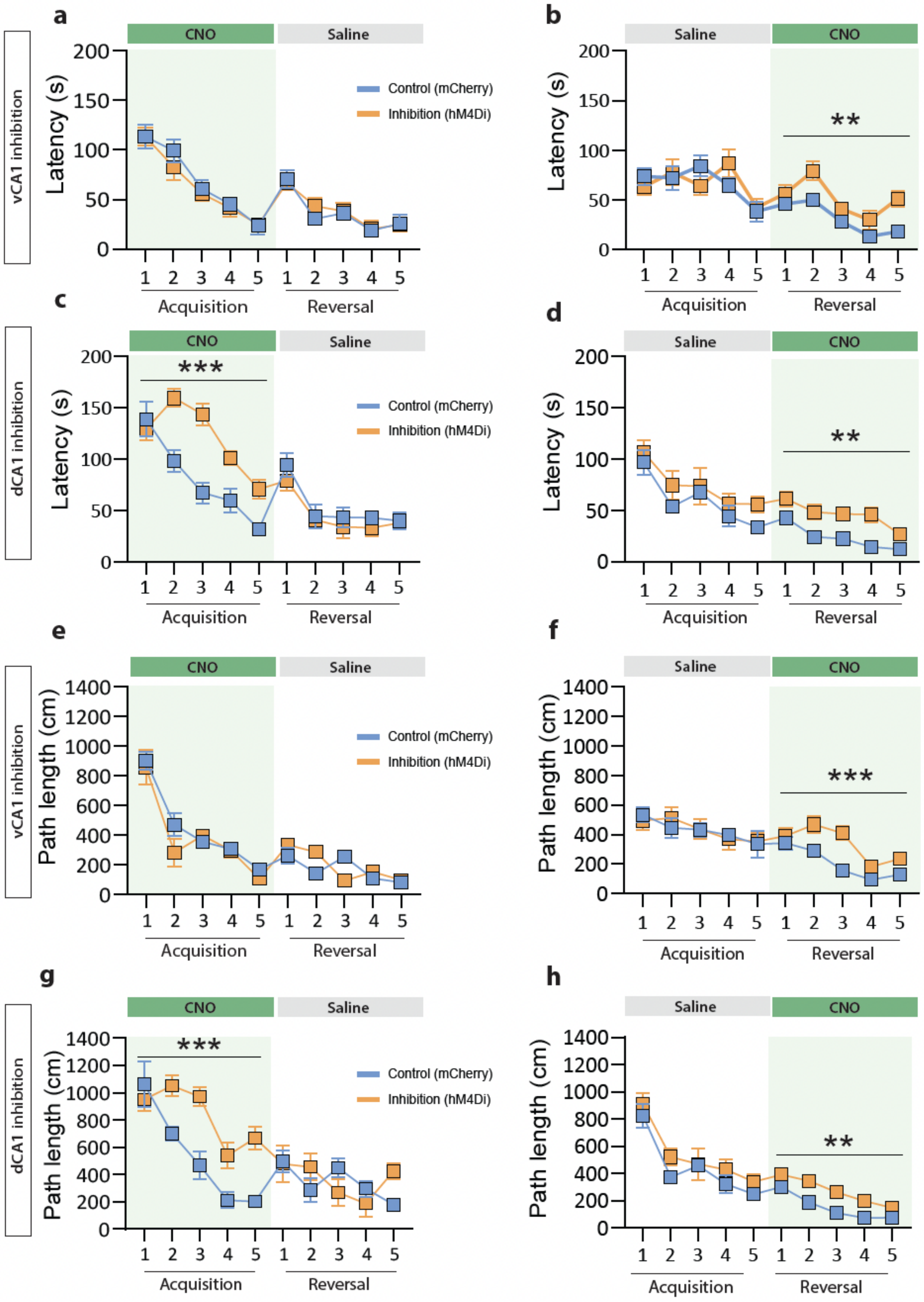

Supplementary Figure 8. Latency and path length to find the escape hole in the Barnes Maze, related to Figure 4. a-d. Latency to find the escape hole. **a.** vCA1 inhibition during acquisition did not affect latency (Two-Way RM ANOVA; Inhibition × Time, F(4,40)= 0.41, P=0.8; Inhibition F(1,10)=0.59, P=0.45; Time F(4,40)=42.89, ***P<0.0001; $n_{control}$=6, n_{hM4D}=6). **b.** vCA1 inhibition after the rule change increased latency (Inhibition × Time, F(4,40)=1.57, P=0.2; Inhibition, F(1,10)=14.10, **P=0.004; Time, F(4,40)=16.79, ***P<0.0001; $n_{control}$=6, n_{hM4D}=6). **c.** dCA1 inhibition during acquisition increased latency (Inhibition × Time, F(4,40)= 4.63, **P=0.003, Inhibition F(1,10)= 43.44, ***P<0.0001; Time F(4,40)=21.07, ***P<0.0001; $n_{control}$=6, n_{hM4D}=6). **d.** dCA1 inhibition after the rule change increased latency (Inhibition × Time, F(4,44)=0.98, P=0.42; Inhibition, F(1,11)=18.51, **P=0.0013; Time F(4,44)=13.20, ***P<0.0001; n_{cont}=6, n_{hM4Di}=7). **e.** vCA1 inhibition during acquisition did not affect path length (Inhibition × Time, F(4,40)=0.87, P=0.48; Inhibition, F(1,10)=2.26, P=0.16: Time, F(4,40)=36.62, ***P<0.0001). **f.** vCA1 inhibition after the rule change increased path length (Inhibition × Time, F(4,40)=2.2, P=0.09; Inhibition F(1,10)=34.14, ***P=0.0002; Time F(4,40)=15.41, ***P<0.0001). **g.** dCA1 inhibition during acquisition increased path length (Inhibition × Time, F(4,40)=3.88, **P=0.009; Inhibition, F(1,10)=27.55, ***P=0.0002; Time, F(4,40)=19.42, ***P<0.0001). **h.** dCA1 inhibition after the rule change increased path length (Inhibition × Time, F(4,44)=0.65, P=0.62; Inhibition, F(1,11)=16.44, **P=0.001; Time, F(4,44)=17.47, ***P<0.0001). Mean±S.E.M.

Supplementary Figure 9.

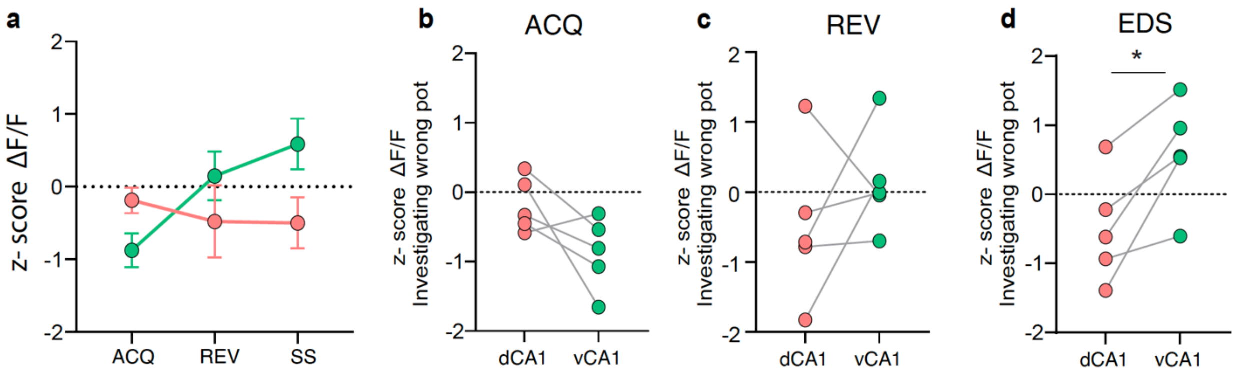

Supplementary Figure 9. Ca^{2+} activity for investigation periods on wrong choice trials, related to Figure 5. a. Average Ca^{2+} activity (z-scored ΔF/F over 3-sec period after investigation onset) for dCA1 and vCA1 on wrong trials on acquisition (ACQ), reversal (REV), and extradimensional set-shift (EDS) days. **b-c.** Pairwise comparison of Ca^{2+} activity showed no significant differences between dCA1 and vCA1 for ACQ and REV days. **d.** vCA1 activity was higher than dCA1 activity during wrong pot investigation on the EDS day (*P=0.02). n=5 mice. Mean±S.E.M.

Supplementary Figure 10.

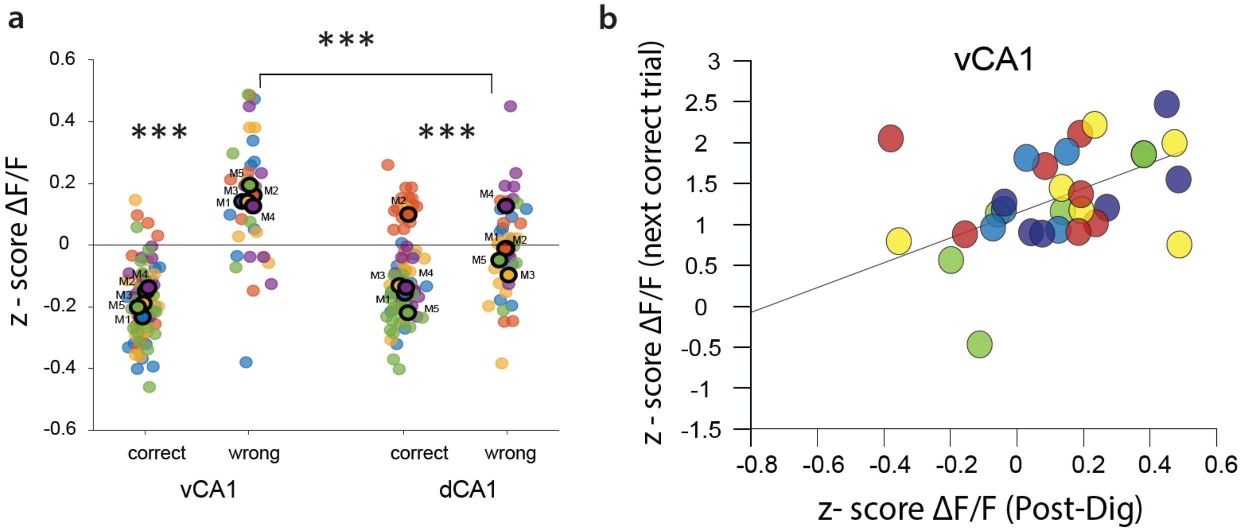

Supplementary Figure 10. Linear Mixed Effects Model accounting for cross-subject variability in trial-level analyses in the flexible odor/texture-based reward association task, related to Figure 5. Trials from each mouse are color-coded. Blue – mouse 1, orange – mouse 2, yellow – mouse 3, purple – mouse 4, green – mouse 5. **a.** Post-dig Ca^{2+} activity (z-scored ΔF/F) for all trials from all mice on the REV day. Linear Mixed Effects Model with 'mouse' as random factor (Choice × Region, β= 0.225, ***P<0.0001; dCA1, β=0.104, ***P<0.0001; vCA, β=0.331, ***P<0.0001). Leave-one-out analysis, confirmed that these effects were not driven by any single mouse, ***P<0.0001 for interaction, *P<0.05 for main effects. n=5 mice. The mean for each mouse is marked with a black outline. **b.** Linear Mixed Effects Model with 'mouse' as random factor revealed a positive relationship between post-dig Ca^{2+} activity (z-scored ΔF/F) and subsequent correct trial Ca^{2+} activity (β = 1.88, ***P<0.0001). Leave-one-out analyses confirmed that this effect remained significant regardless of which mouse was excluded (all *P<0.05).

Supplementary Figure 11.

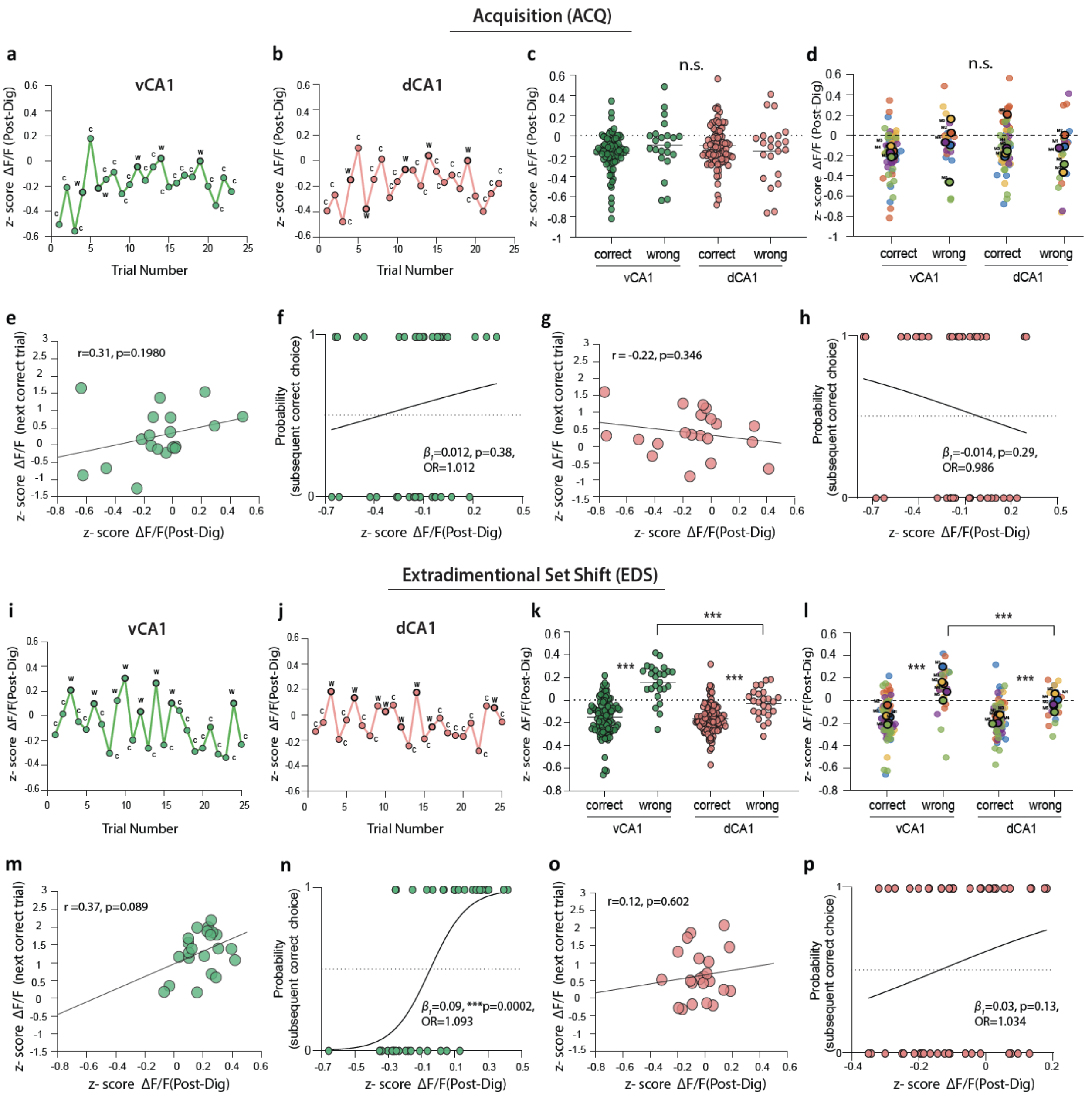

Supplementary Figure 11. Perseverative error signals in vCA1 and dCA1 on ACQ and EDS days do not predict subsequent activity or choices, related to Figure 5. a-h. Acquisition Day (ACQ). **a.** Representative example of vCA1 Ca^{2+} activity (z-scores) during 3-sec post-digging epochs from one mouse across trials on the ACQ day. w – post-dig Ca^{2+} activity after wrong choices; c – post-dig Ca^{2+} activity after correct choices. Post-dig activity on error trials that are followed by a correct choice are outlined. **b.** Representative example of dCA1 Ca^{2+} activity during

post-digging epochs from one mouse across trials on the ACQ day. w –wrong choices; c –correct choices. Post-dig activity on error trials that are followed by a correct choice are outlined. **c.** No differences between post-dig Ca^{2+} activity for all trials from all mice on the ACQ day (Choice × Region, $F(1,200)=2.39$, $P= 0.12$; Choice, $F(1,200)=0.13$, $P=0.72$; Region, $F(1,200)=0.02$, $P=0.89$). **d.** Representation of trials from each mouse with color-coding: Blue – mouse 1, orange – mouse 2, yellow – mouse 3, purple – mouse 4, green – mouse 5. The mean of each mouse is marked with a black outline. **e.** vCA1 post-dig neural activity did not correlate with neural activity during investigation on subsequent correct trials ($r=0.31$, $P=0.198$). **f.** Logistic regression model shows no significant relationship between vCA1 post-dig activity and the probability of making a correct choice on the next trial ($\beta_1=0.012$, $|Z|=0.87$, $P=0.038$, OR=1.012). **g.** dCA1 post-dig activity did not correlate with activity during investigation on subsequent correct trials ($r=-0.22$, $P=0.346$). **h.** Logistic regression model shows no significant relationship between dCA1 post-dig activity and the probability of making a correct choice on the next trial ($\beta_1=0.014$, $|Z|=1.07$, $P=0.29$, OR=0.986). **i-p.** Extradimensional Set Shift Day (EDS). **i.** Representative example of vCA1 Ca^{2+} activity (z-scores) during 3-sec post-digging epochs from one mouse across trials on the EDS day. w – post-dig Ca^{2+} activity after wrong choices; c – post-dig Ca^{2+} activity after correct choices. Post-dig activity on error trials that are followed by a correct choice are outlined. **j.** Representative example of dCA1 Ca^{2+} activity during post-digging epochs from one mouse across trials on the EDS day. w –wrong choices; c –correct choices. Post-dig activity on error trials that are followed by a correct choice are outlined. **k.** Post-dig neural activity after wrong trials (error signals) was higher in vCA1 than in dCA1 (Choice × Region, $F(1,242)=14.08$, $***P=0.0002$; Choice, $F(1,242)=93.54$, $***P<0.0001$; Region, $F(1,242)=18.79$, $P<0.0001$; $vCA1_{wrong\ choice}$ vs $dCA1_{wrong\ choice}$, $***P<0.0001$). **l.** Representation of trials from each mouse with color-coding: Blue – mouse 1, orange – mouse 2, yellow – mouse 3, purple – mouse 4, green – mouse 5. The mean of each mouse is marked with a black outline. Linear Mixed Effects Model with 'mouse' as random factor (Choice × Region, $\beta=0.225$, $***P<0.0001$; dCA1, $\beta=0.104$, $***P<0.0001$; vCA, $\beta=0.331$, $***P<0.0001$). Leave-one-out analysis, $***P<0.0001$ for interaction, $*P<0.05$ for main effects. n=5 mice. **m.** vCA1 post-dig neural activity did not correlate with neural activity during investigation on subsequent correct trials ($r=0.37$, $P=0.089$). **n.** Logistic regression model shows a significant relationship between vCA1 post-dig activity and the probability of making a correct choice on the next trial ($\beta_1=0.09$, $|Z|=3.76$, $***P=0.0002$, OR=1.093). **o.** dCA1 post-dig activity did not correlate with activity during investigation on subsequent correct trials ($r=0.12$, $P=0.602$). **p.** Logistic

regression model shows no significant relationship between dCA1 post-dig activity and the probability of making a correct choice on the next trial (β_1=0.03, |Z|=1.53, P=0.13, OR=1.034).

Supplementary Figure 12.

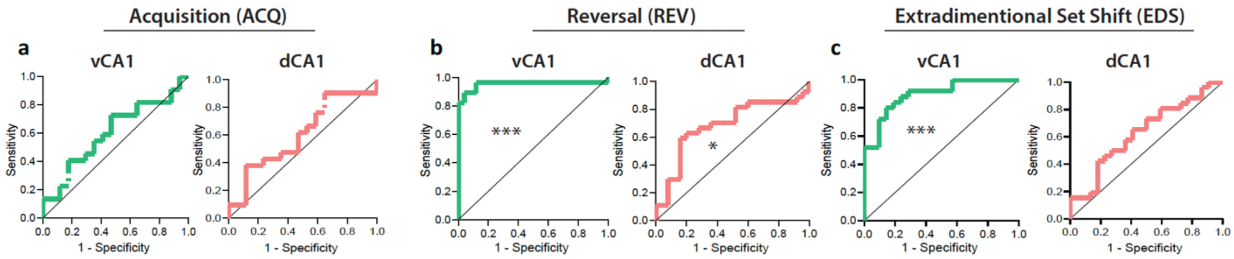

Supplementary Figure 12. Receiver Operating Characteristic (ROC) curve shows performance of logistic regression models to predict choice behavior from post-dig neural activity, related to Figure 5. a. ROC curve for logistic regression model performance on Acquisition Day (ACQ). Left: vCA1 (AUC=0.6±0.09, P=0.27), Right: dCA1 (AUC=0.6±0.09, P=0.28). **b.** ROC curve for Reversal Day (REV). Left: vCA1 (AUC=0.95±0.04, ***P<0.0001), Right: dCA1 (AUC=0.68±0.08, *P=0.02). **c.** ROC curve for Extradimensional Set Shift Day (EDS). Left: vCA1 (AUC=0.9±0.05, ***P<0.0001), Right: dCA1 (AUC=0.6±0.08, P=0.12).
